# Myeloid‐Derived Suppressor Cell Accumulation Drives Intestinal Fibrosis through mCCL6/hCCL15 Chemokine‐Mediated Fibroblast Activation

**DOI:** 10.1002/advs.202411711

**Published:** 2024-12-31

**Authors:** Xiaohui Cheng, Pingwen Shao, XinTong Wang, Juan Jiang, Jiahui Chen, Jie Zhu, Weiming Zhu, Yi Li, Junfeng Zhang, Jiangning Chen, Zhen Huang

**Affiliations:** ^1^ State Key Laboratory of Pharmaceutical Biotechnology School of Life Sciences Nanjing University Nanjing Jiangsu 210023 China; ^2^ Department of General Surgery Jinling Hospital School of Medicine Nanjing University Nanjing Jiangsu 210002 China; ^3^ State Key Laboratory of Analytical Chemistry for Life Sciences Nanjing University Nanjing Jiangsu 210023 China; ^4^ NJU Xishan Institute of Applied Biotechnology Xishan District Wuxi Jiangsu 214101 China

**Keywords:** IBD, intestinal fibroblasts, intestinal fibrosis, mCCL6/hCCL15, MDSCs

## Abstract

Intestinal fibrosis, a severe complication of Crohn's disease (CD), is linked to chronic inflammation, but the precise mechanism by which immune‐driven intestinal inflammation leads to fibrosis development is not fully understood. This study investigates the role of myeloid‐derived suppressor cells (MDSCs) in intestinal fibrosis in CD patients and a 2,4,6‐trinitrobenzene sulfonic acid (TNBS)‐induced mouse model. Elevated MDSCs are observed in inflamed intestinal tissues prior to fibrosis and their sustained presence in fibrotic tissues of both CD patients and murine models. Depletion of MDSCs significantly reduces fibrosis, highlighting their key role in the fibrotic process. Mechanistically, MDSC‐derived mCCL6 activates fibroblasts via the CCR1‐MAPK signaling, and interventions targeting this axis, including neutralizing antibodies, a CCR1 antagonist, or fibroblast‐specific *Ccr1* knockout mice reduce fibrosis. In CD patients with stenosis, human CCL15, analogous to mCCL6, is found to be elevated in MDSCs and activated fibroblasts. Additionally, CXCR2 and CCR2 ligands are identified as key mediators of MDSC recruitment in intestinal fibrosis. Blocking MDSC recruitment with CXCR2 and CCR2 antagonists alleviates intestinal fibrosis. These findings suggest that strategies targeting MDSC recruitment and mCCL6/hCCL15 signaling could offer therapeutic benefits for intestinal fibrosis.

## Graphical abstract

In experimental models of intestinal fibrosis and Crohn's disease (CD) patients with stenosis, elevated CXCR2 and CCR2 ligands recruit myeloid‐derived suppressor cells (MDSCs). These MDSCs secrete mCCL6 and hCCL15, which activate fibroblasts and drive fibrosis. Therefore, strategies aimed at inhibiting MDSC accumulation and blocking the mCCL6/hCCL15‐mediated signaling pathways represent promising approaches for the management of fibrotic conditions in CD.

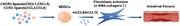

## 1 Introduction

Intestinal fibrosis is a prevalent and challenging complication associated with inflammatory bowel diseases (IBD), with particular relevance to Crohn's disease (CD). It frequently results in intestinal lumen narrowing, precipitating stenosis or even complete obstruction.^[^ ^1^ ^]^ The pathogenesis of CD‐related fibrosis is fundamentally linked to chronic intestinal inflammation, which initiates a vicious cycle of tissue damage and repair. This cycle ultimately leads to the activation and proliferation of intestinal fibroblasts, culminating in the pathological accumulation of extracellular matrix (ECM).^[^ ^2^, ^3^ ^]^ Conventional therapeutic approaches, including endoscopic interventions and surgical resection, offer only temporary relief due to the propensity for fibrosis to recur. Although advanced biologic therapies have shown efficacy in reducing inflammation, their capacity to resolve established fibrosis is considerably limited.^[^ ^4^, ^5^, ^6^ ^]^ The progression of intestinal fibrosis is intimately linked to mucosal inflammation, however, the comprehension of immune cell dynamics and the functions of their secreted cytokines during fibrosis development remains incomplete. Immunoregulatory cells and their derived factors, which have the potential to alleviate colitis, play complex roles in the development of intestinal fibrosis. For instance, immunosuppressive cells such as regulatory T cells (Tregs) typically mitigate inflammation but can exert paradoxical effects in fibrotic diseases.^[^ ^7^, ^8^, ^9^, ^10^ ^]^ Furthermore, immunoregulatory factors like TGF‐β1 can suppress inflammation while simultaneously accelerating fibrosis.^[^ ^11^, ^12^ ^]^ Therefore, there is a growing interest in the role of these immunosuppressive cell populations in the pathogenesis of fibrosis.

Myeloid‐derived suppressor cells (MDSCs), a heterogeneous group of immature myeloid cells, have emerged as central players in various pathological states, ranging from malignancies to chronic inflammatory conditions.^[^ ^13^, ^14^ ^]^ While MDSCs are known for their ability to suppress T cell activity and promote immune evasion in cancer, their role in intestinal inflammation remains is less understood and is complicated by conflicting reports regarding their immunoregulatory functions.^[^ ^15^, ^16^, ^17^, ^18^ ^]^ Additionally, MDSC‐associated cytokines, such as TGF‐β1 and IL‐10, exhibit diverse effects across different fibrotic diseases, suggesting that the contribution of MDSCs to fibrosis may significantly differ between organs.^[^ ^19^, ^20^ ^]^ This variability implies that the functions of MDSCs in fibrosis are organ‐system specifics. Therefore, a nuanced investigation into the roles of MDSCs in intestinal fibrosis is necessary.

In this study, we have uncovered previously unexplored mechanisms by which MDSCs serve as key facilitators of intestinal fibrosis in CD. Our analysis of immune cell populations in CD patients and 2,4,6‐trinitrobenzene sulfonic acid (TNBS)‐induced mouse models has demonstrated a significant increase in MDSCs in inflamed intestinal tissues prior to the onset of fibrosis and a sustained presence in fibrotic tissues. Through adoptive cell transfer and MDSC deletion experiments, we have established that MDSCs actively promote the development of intestinal fibrosis, rather than being a mere consequence of the fibrotic process. Our mechanistic insights reveal that MDSC‐derived mouse C─C motif ligand 6 (mCCL6) activates intestinal fibroblasts via the CCR1‐MAPK pathway, leading to excessive extracellular matrix (ECM) deposition. Similarly, the human homolog of mCCL6, human C─C motif ligand 15 (hCCL15), also activates myofibroblast CCD18Co cells. Ligands for CCR2 and CXCR2, including CCL2, CCL8, CXCL1, and CXCL5, are responsible for the recruiting MDSCs into fibrotic tissues. Collectively, these findings suggest that targeting MDSC recruitment or the mCCL6/hCCL15 pathway may represent a promising therapeutic strategy for CD‐induced fibrosis.

## 2 Results

### 2.1 MDSCs Significantly Increased in Colon Tissues of the TNBS‐Induced Intestinal Fibrosis Model

**Figure 1:**
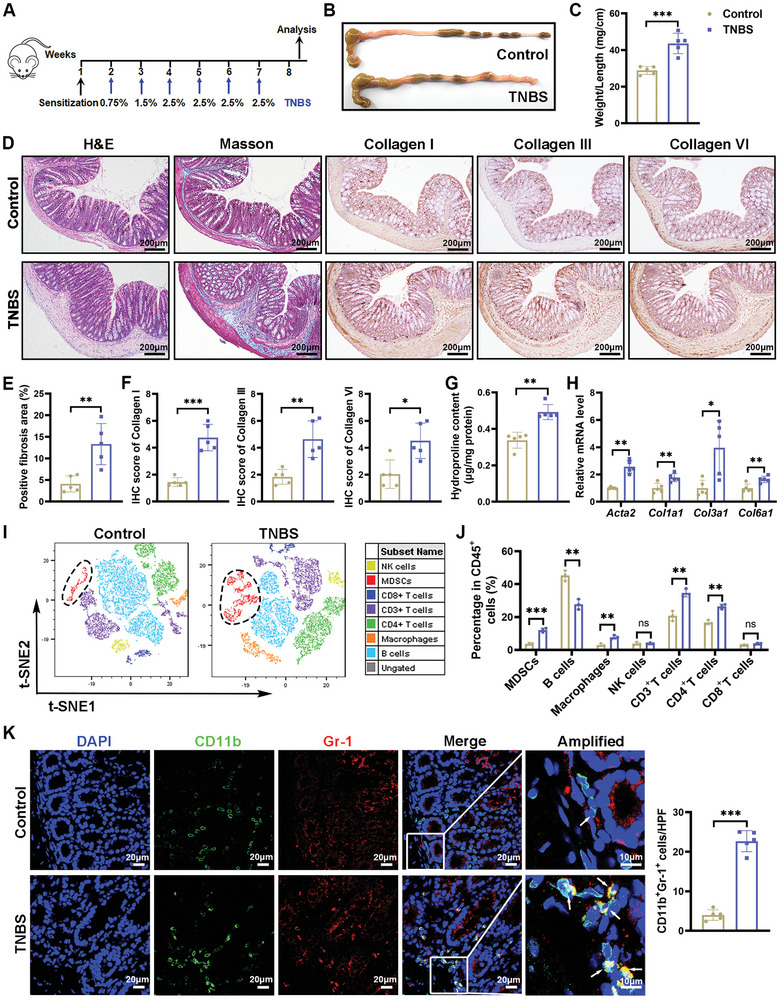
Induction of intestinal fibrosis in mice through repeated intracolonic administration of TNBS resulted in an increased proportion of MDSCs. A) The schematic diagram depicts the methodology for the establishment of TNBS‐induced intestinal fibrosis in mice. B,C) Eight weeks post‐initial TNBS treatment, colon tissues were harvested for gross examination and the calculation of the weight‐to‐length ratio. D) Histological examination, including hematoxylin & eosin (H&E) staining, Masson's trichrome staining, and immunohistochemical (IHC) staining for collagen types I, III and VI was performed on serial sections of colon tissue from both control and TNBS‐treated mice. Scale bar = 200 µm. E) The mean percentage of areas positive for Masson's trichrome and F) the semiquantitative scoring of IHC staining images were performed using ImageJ software. Extracellular matrix deposition in the colon tissues was measured by G) hydroxyproline assay and H) qRT‐PCR analyses for *Acta2*, *Col1a1*, *Col3a1*, and *Col6a1*. *n* = 5 mice per group for (C–H). I,J) Flow cytometry was applied to identify the immune cell subsets within the colonic lamina propria, with data visualized using t‐SNE maps and histograms. *n* = 3 mice per group. K) Immunofluorescence staining visualized MDSCs (CD11b^+^Gr‐1^+^) in the colon tissues. Red, Gr‐1; green, CD11b; blue, DAPI for nuclear staining. Scale bars are provided in the images. *n* = 5 mice each group. D–F,K) Tissue sections from each mouse were examined, and five fields were captured for quantitative analysis, with each data point representing the mean of these fields. Data are presented as means ± SD. Statistical analyses were performed using an unpaired t‐test with Welch's correction (two tailed) for (H) (*Acta2*, *Col3a1*), Mann‐Whitney U test for (G), and unpaired Student's t‐test (two tailed) for other panels. *p < 0.05, **p < 0.01 and ***p < 0.001, ns, not significant.

The intestinal fibrosis model was established following the protocol outlined in **Figure** **1**A. Macroscopic examination showed that TNBS‐treated mice had significantly thicker colons and a higher weight‐to‐length ratio compared to healthy controls (Figure 1B,C). Histological analysis revealed critical features of intestinal fibrosis, such as submucosal thickening and muscular layer hyperplasia. The presence of Masson's trichrome‐positive blue areas, combined with immunohistochemical staining for Collagen I/III/VI and elevated hydroxyproline levels, confirmed increased collagen deposition in the colon tissues of TNBS‐treated mice (Figure 1D–G). Furthermore, qRT‐PCR analysis demonstrated significantly elevated mRNA expression levels of *Acta2*, *Col1a1*, *Col3a1*, and *Col6a1* in the fibrotic colon tissues compared to normal controls (Figure 1H). These findings strongly suggest that this mouse model closely mirrors the pathology of human intestinal fibrosis.

Flow cytometry was conducted to assess immune cell infiltration in the colonic lamina propria following TNBS treatment (Figure 1I; Figure S1, Supporting Information). T‐SNE dimensionality reduction was applied for statistical analysis. The results revealed a significant increase in the proportions of MDSCs, macrophages, and CD4^+^ T cells, accompanied by a decrease in B cell proportions. However, the ratios of CD8^+^ T cells, NK cells and Treg cells remained unchanged in TNBS‐treated mice compared to normal controls (Figure 1J and Figure S2, Supporting Information). Immunofluorescence analysis further confirmed the increased presence of MDSCs in the colonic lamina propria of TNBS‐treated mice (Figure 1K). Notably, both monocytic MDSCs (M‐MDSCs, CD11b^+^Gr‐1^+^Ly6C^+^) and polymorphonuclear MDSCs (PMN‐MDSCs, CD11b^+^Gr‐1^+^Ly6G^+^) subtypes were elevated in the colons of TNBS‐treated mice, with a more pronounced increase observed in M‐MDSCs (Figure S3A, Supporting Information). Interestingly, even in the early stages of intestinal fibrosis, when fibrosis symptoms are not yet apparent, MDSC proportions were already elevated (Figure S4A,B, Supporting Information). This persistent increase in MDSCs suggests a strong correlation with the progression of intestinal fibrosis.

### 2.2 MDSCs Exhibited Abnormal Accumulation in the Lamina Propria of CD Patients during the Progression of Intestinal Fibrosis

**Figure 2:**
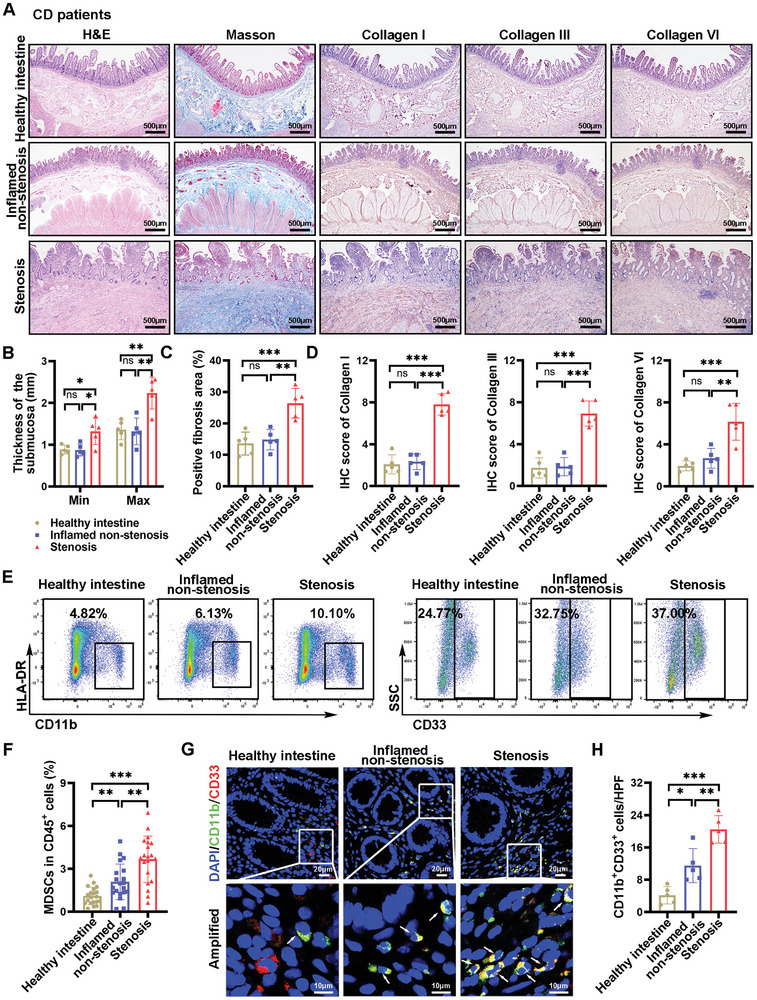
Abnormal accumulation of MDSCs in the lamina propria of Crohn's disease (CD) patients during the progression of intestinal fibrosis. A) Histological examination, including hematoxylin & eosin (H&E) staining, Masson's trichrome staining, and immunohistochemical staining for collagen types I, III and VI, was performed on serial intestinal tissue sections from different regions of CD patients. B–D) The ImageJ software was used to quantify the minimum and maximum submucosal thickness, the percentage of areas positive for Masson's trichrome, as well as the IHC staining scores (*n* = 5 patients). E,F) Flow cytometry was employed to detect the proportions of MDSCs (CD33^+^CD11b^+^HLA‐DR^−/low^) in different intestine segments of CD patients, and statistical analysis was performed (*n* = 20 patients). G,H) Immunofluorescence staining was conducted to visualize MDSCs (CD11b^+^CD33^+^) in different segments from CD patients, and the number of positive cells was quantified. The colors represent: red, CD33; green, CD11b; blue, DAPI nuclear staining. A scale bar is included in the image for reference (*n* = 5 patients). A,B,G) Tissue sections from each patient were examined, and five fields were captured for quantitative analysis, with each data point representing the mean of these fields. Results are presented as means ± SD. Statistical analyses were performed using Brown‐Forsythe and Welch ANOVA with Tamhane's multiple comparisons test for (F) and one‐way ANOVA with Bonferroni's multiple comparison test for the other panels. *p < 0.05, **p < 0.01 and ***p < 0.001, ns, not significant.

A pathological examination was conducted to investigate the characteristics of different intestinal segments in patients with CD and intestinal stenosis. H&E and Masson's trichrome staining revealed marked submucosal thickening and increased collagen deposition in the stenotic intestinal tissues of CD patients, compared to healthy and inflamed non‐stenosis tissues (**Figure** **2**A–C). Immunohistochemical staining for collagen types I, III, and VI confirmed substantial collagen accumulation in the mucosal and submucosal layers of stenotic tissues in CD patients (Figure 2A,D). Flow cytometry was utilized to assess the ratios of MDSCs, characterized as CD11b^+^CD33^+^HLA‐DR^−/low^, and other immune cell types in healthy intestine, inflamed non‐stenosis tissues, and stenosis tissue (Figure S5, Supporting Information). The results showed that the proportion of MDSCs was elevated in inflamed non‐stenotic tissues and further increase in stenotic tissues (Figure 2E,F). Immunofluorescence analysis corroborated these findings, showing a progressive increase in CD11b/CD33 double‐positive cells (MDSCs) from healthy to stenotic tissues in CD patients (Figure 2G,H). Subtype analysis identified CD11b^+^CD33^+^HLA‐DR^−/low^CD14^+^ MDSCs (M‐MDSCs) and CD11b^+^CD33^+^HLA‐DR^−/low^CD15^+^ MDSCs (PMN‐MDSCs) as contributors to the overall MDSC increase in intestinal stenosis samples (Figure S3B, Supporting Information). Additionally, flow cytometry analysis revealed an elevation in B cell populations, though the patterns varied among species. While CD4^+^ T cells and macrophages were also increased in stenotic tissues, they did not exhibit the same progressive increase observed in MDSCs. CD8^+^ T cells decreased in inflamed non‐stenotic tissues and did not correlate positively with the severity of fibrosis (Figure S6, Supporting Information). These observations highlight MDSCs as a key subset of immune cells potentially driving the progression of intestinal fibrosis, prompting further investigation into their role.

### 2.3 MDSCs Promoted the Progression of Intestinal Fibrosis

**Figure 3:**
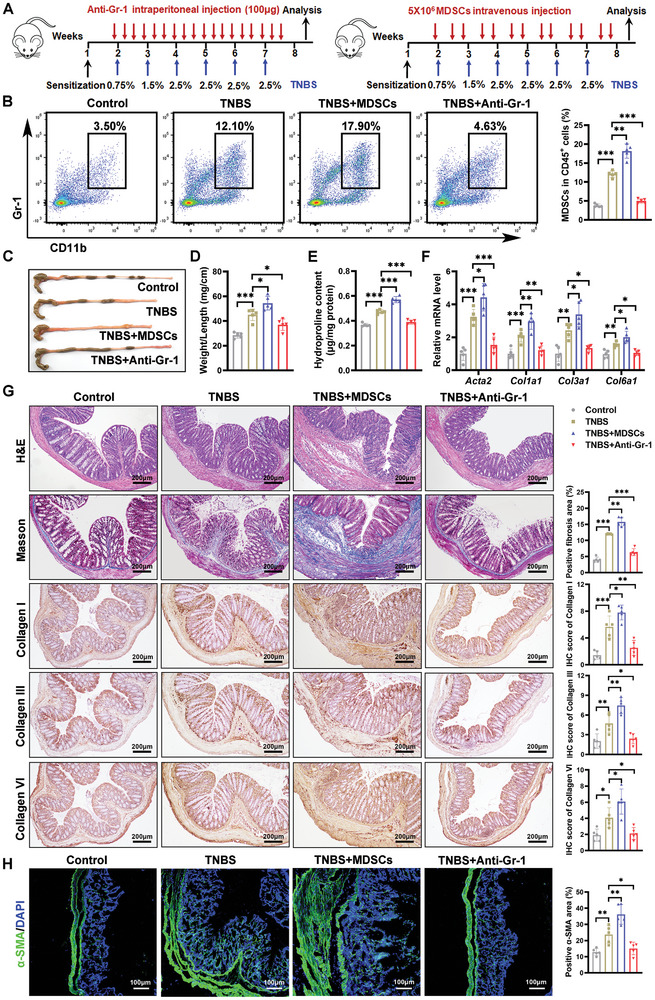
The impact of adoptive transfer of MDSCs or depletion MDSCs via anti‐Gr‐1 antibody on the TNBS‐induced mouse model of intestinal fibrosis was investigated. A) Experimental design for in vivo depletion and adoptive transfer of MDSCs in the intestine fibrosis mouse model is depicted. B) Flow cytometry was conducted to evaluate the MDSC population within the colonic lamina propria of mice subjected to various treatments. Colon tissues were harvested for C) morphological assessment, D) calculation of the weight‐to‐length ratio, E) hydroxyproline assay, and F) qRT‐PCR analysis of fibrotic markers (*Acta2*, *Col1a1*, *Col3a1*, and *Col6a1*). Histological evaluations, including G) H&E staining, Masson's trichrome staining, immunohistochemical staining for collagen types I, III, and VI, as well as H) immunofluorescence staining for α‐SMA, were performed on tissue sections from control and TNBS‐treated mice receiving different treatments. The quantification of Masson's trichrome and α‐SMA‐positive areas, along with IHC staining scores, was carried out using ImageJ software. Green, α‐SMA; blue, DAPI for nuclear staining. A scale bar is provided for reference. *n* = 5 mice per group. G,H) Tissue sections from each mouse were examined, and five fields were captured for quantitative analysis, with each data point representing the mean of these fields. Data are presented as means ± SD. Statistical analyses were performed using Brown‐Forsythe and Welch ANOVA with Tamhane's multiple comparisons test for (B,G) (analysis of Masson's trichrome staining), and one‐way ANOVA with Bonferroni's multiple comparison test for the remaining panels. *p < 0.05, **p < 0.01 and ***p < 0.001, ns, not significant.

To investigate the role of MDSCs in intestinal fibrosis, we modulated MDSC levels in TNBS‐induced fibrotic mice using anti‐Gr‐1 antibody treatment and MDSC adoptive transfer (**Figure** **3**A). An intraperitoneal injection of the anti‐Gr‐1 antibody effectively depleted colonic MDSCs for 48 h with minimal impact on other immune cells, including T cells, B cells, macrophages, and NK cells (Figure S7, Supporting Information). This result confirms the efficiency and specificity of MDSCs depletion via anti‐Gr‐1 antibody treatment. As shown in Figure 3B, repeated treatments of the anti‐Gr‐1 antibody maintained reduced MDSC levels in the colonic lamina propria, whereas MDSC adoptive transfer significantly increased MDSC proportions. Mice that received MDSC adoptive transfer exhibited an increased colon diameter (Figure 3C), higher weight‐to‐length ratio (Figure 3D), elevated hydroxyproline levels (Figure 3E), and upregulated expression of fibrotic markers *Acta2*, *Col1a1*, *Col3a1*, and *Col6a1* (Figure 3F). Conversely, anti‐Gr‐1 antibody treatment had the opposite effects. Histopathological analysis revealed that MDSC adoptive transfer led to increased Masson's trichrome staining and collagen content within the colons, whereas anti‐Gr‐1 antibody treatment resulted in reduced ECM deposition and fewer activated fibroblasts (Figure 3G,H). These results underscore the critical role of MDSCs in the development of intestinal fibrosis and suggest that their depletion could potentially mitigate disease progression.

### 2.4 MDSCs Promoted Fibroblasts Activation and Collagen Production in Intestine Fibrosis

To investigate the mechanisms underlying the contribution of MDSCs to intestinal fibrosis, we conducted a series of in vitro experiments. While previous studies have suggested that MDSCs may differentiate into fibrocytes, which are implicated in intestinal stenosis,^[^ ^21^, ^22^ ^]^ our flow cytometry analysis did not detect significant changes in the proportion of fibrocytes (CD45 and type I collagen double‐positive cells) in the colons of TNBS‐treated mice with intestinal fibrosis compared to healthy controls (Figure S8, Supporting Information). We also examined whether MDSCs could induce epithelial‐to‐mesenchymal transition (EMT), a process critical for intestinal fibrosis.^[^ ^23^ ^]^ However, co‐culturing colonic MDSCs from TNBS‐treated mice with MC38 cells did not significantly alter EMT markers, including *Cdh1*, *Acta2*, *Vimentin*, *Snail*, and *Fsp1* (Figure S9, Supporting Information).

**Figure 4:**
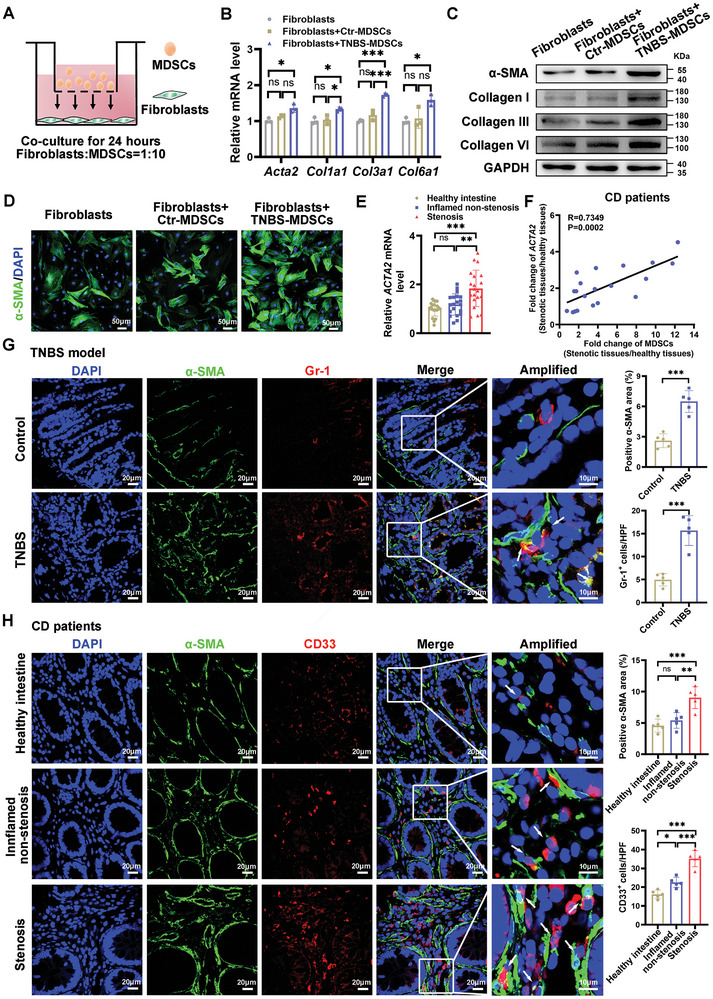
MDSCs from the colon promoted fibroblasts activation and collagen production in intestinal fibrosis. A) Schematic illustration of the co‐culture setup with primary colonic MDSCs and intestinal fibroblasts at a ratio of 10:1 for 24 h. B,C) qRT‐PCR and western blotting analysis were used to assess the mRNA and protein levels of α‐SMA and collagen (types I, III, and VI) in fibroblasts following co‐culture with MDSCs. D) Immunofluorescence staining for α‐SMA in cocultured fibroblasts is shown. Green, α‐SMA; blue, DAPI for nuclear staining. Images are representative of three independent experiments. *n* = 3 biologically independent samples for (B), and the experiments were repeated three times independently with similar results for (D). E) mRNA levels of *ACTA2* in healthy, inflamed non‐stenotic and stenotic intestinal tissues from CD patients were analyzed by qRT‐PCR (*n* = 20 patients). F) Correlation analysis between the relative expression of *ACTA2* and MDSC levels in stenotic tissues versus healthy tissues from CD patients (*R* = 0.7349, *P* = 0.0002, *n* = 20 patients). G) Double‐staining for Gr‐1 and α‐SMA in colon tissues from TNBS‐treated mice is depicted, with boxed areas highlighting the proximity of Gr‐1^+^ and α‐SMA^+^ cells. *n* = 5 mice each group. H) Double‐stained for CD33 and α‐SMA in stenotic, inflamed non‐stenotic, and healthy intestinal tissues from CD patients is shown, with boxed areas indicating the proximity of CD33^+^ and α‐SMA^+^ cells. *n* = 5 patients. Green, α‐SMA; red, CD33 or Gr‐1; blue, DAPI for nuclear staining. Scale bars are included in (D,G,H) for reference. G,H) Tissue sections from each mouse or patient were examined, and five fields were captured for quantitative analysis, with each data point representing the mean of these fields. Data are presented as means ± SD. Statistical analyses were performed using Kruskal‐Wallis test with Dunn's multiple comparisons test in (B) (*Acta2*), Brown‐Forsythe and Welch ANOVA with Tamhane's multiple comparisons test for (E), Pearson's correlation analysis for (F), unpaired Student's t test (two tailed) for (G), and one‐way ANOVA with Bonferroni's multiple comparison test for other panels. *p < 0.05, **p < 0.01 and ***p < 0.001, ns, not significant.

We subsequently examined the effect of MDSCs on intestinal fibroblasts using a transwell‐based co‐culture model (**Figure** **4**A). The optimal co‐culture ratio (fibroblasts: MDSCs = 1:10) was determined based on *Acta2* levels in fibroblasts (Figure S10, Supporting Information). In these co‐culture experiments, MDSCs derived from TNBS‐induced intestinal fibrosis mice stimulated the activation of colonic fibroblasts and enhanced collagen production, as evidenced by increased expression of α‐SMA and collagen types I, III, and VI in fibroblasts treated with MDSCs (Figure 4B–D). In clinical samples, *ACTA2* expression was significantly higher in intestinal stenosis tissues compared to normal and inflamed non‐stenotic tissues from CD patients (Figure 4E). Moreover, a positive correlation was observed between the fold changes in MDSC levels and the increase in *ACTA2* expression in stenotic sites compared to healthy regions of CD patients (Figure 4F). Immunofluorescence analysis of colon tissues from TNBS‐induced intestinal fibrosis mice revealed significant increases in both the α‐SMA‐positive area and the number of Gr‐1^+^ cells (Figure 4G). Similarly, in intestinal stenotic sites of CD patients, both the α‐SMA‐positive area and the number of CD33^+^ cells were significantly elevated. Moreover, CD33^+^ MDSCs were often found in close proximity to α‐SMA^+^ spindle cells (Figure 4H). These findings strongly support the concept that MDSCs promote the activation of fibroblasts and enhance collagen production through interactions with intestinal fibroblasts, thereby contributing to the development of intestinal fibrosis.

### 2.5 MDSCs Induced Fibroblasts Activation and Collagen Production through the Release of mCCL6/hCCL15

**Figure 5:**
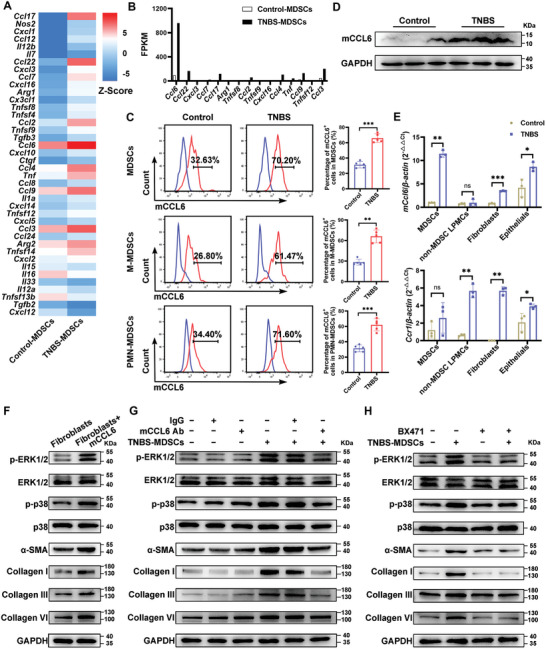
MDSCs‐derived mCCL6 activated fibroblasts and promoted collagen production. A) A heatmap illustrates the differential expression of cytokines and chemokines in colonic MDSCs from control and TNBS‐treated intestinal fibrosis mice. B) Sequence reads for these cytokines and chemokines in colonic MDSCs isolated from normal and TNBS‐induced intestinal fibrosis mice are presented. C) Flow cytometry was performed to assess mCCL6 expression in colonic lamina propria MDSCs, including M‐MDSCs and PMN‐MDSCs, from control and TNBS‐treated intestinal fibrosis mice (*n* = 5 mice per group). D) Western blotting was used to assess mCCL6 protein levels in colons from control and TNBS‐treated intestinal fibrosis mice. E) qRT‐PCR was used to evaluate mRNA levels of m*Ccl6* and *Ccr1* in epithelial cells, fibroblasts, MDSCs, and non‐MDSC LPMCs within the lamina propria (*n* = 3 mice per group for D–E). F) Primary intestinal fibroblasts were treated with 25 ng mL^−1^ mCCL6 to assess the levels of phosphorylated ERK1/2 (p‐ERK1/2) and phosphorylated p38 (p‐p38) at 30 min post‐stimulation, and α‐SMA, collagen I, collagen III, and collagen VI at 24 h post‐stimulation. G,H) Primary intestinal fibroblasts were co‐cultured with fibrotic colonic MDSCs at a ratio of 1:10. In selected experiments, mCCL6‐neutralizing antibodies (40 ng mL^−1^) or BX471 (20 µm) were added to the co‐culture. Western blotting was used to measure the expression of MAPK pathway molecules and activation markers in the fibroblasts at the indicated time points (p‐ERK1/2 and p‐p38 at 30 min; α‐SMA, collagen I, collagen III, and collagen VI at 24 h). Data are presented as means ± SD. Statistical analyses were performed using the Mann‐Whitney U test for (C) (mCCL6^+^ cells in M‐MDSCs), unpaired t test with Welch's correction (two tailed) for (E) (*mCcl6* expression in MDSCs, *Ccr1* expression in fibroblasts and other LPMCs), and unpaired Student's t test (two tailed) for other panels. *p < 0.05, **p < 0.01 and ***p < 0.001, ns, not significant.

Our study demonstrates that MDSCs can activate fibroblasts through indirect interactions, suggesting that MDSC‐derived cytokines play a crucial role in this process. Transcriptome sequencing identified mCCL6 as the predominant cytokine in colonic MDSCs from TNBS‐induced mice, with a significant upregulation (**Figure** **5**A,B). Flow cytometry confirmed the elevated expression of mCCL6 in MDSCs from the colons of TNBS model mice, observed in both M‐MDSCs and PMN‐MDSCs subpopulations (Figure 5C). Additionally, colon tissues from TNBS‐induced intestinal fibrosis mice exhibited aberrant mCCL6 expression levels (Figure 5D). We further isolated MDSCs, other lamina propria mononuclear cells (LPMCs) excluding MDSCs, fibroblasts, and epithelial cells from the colons of both normal and fibrotic mice. Analysis of MDSC‐related cytokines and receptors involved in fibrotic diseases revealed that mCCL6 expression was most significantly increased in MDSCs compared to other cell populations. Moreover, its receptor, CCR1, was highly expressed across various cell types, including LPMCs, fibroblasts, and epithelial cells (Figure 5E). Regarding cytokines TGF‐β1 and IL‐10, which are associated with MDSCs in lung and liver fibrosis,^[^ ^19^, ^20^ ^]^ our measurements showed no significant changes in colonic MDSCs from TNBS‐induced fibrotic mice compared to normal mice (Figure S11, Supporting Information). Based on these findings, we focused on investigating the role and mechanism of MDSC‐derived mCCL6 in the progression of intestinal fibrosis.

Previous studies indicate that mCCL6 activates MAPK signaling pathways (ERK and p38) through the CCR1 receptor, which are central to intestinal fibrosis.^[^ ^24^, ^25^, ^26^, ^27^ ^]^ In this study, treating intestinal fibroblasts with mCCL6 led to increased phosphorylation of ERK1/2 and p38, along with elevated α‐SMA and collagen (types I, III, and VI) expression (Figure 5F; Figure S12A,B, Supporting Information). To further explore the role of MDSC‐derived mCCL6, we used a neutralizing antibody or the CCR1 antagonist BX471 in co‐cultures. These interventions blocked MDSC‐induced fibroblast activation and collagen synthesis (Figure 5G,H; Figure S12C–F, Supporting Information). Human CCL15, homologous to mouse CCL6, was significantly elevated in MDSCs from both inflamed non‐stenotic and stenotic intestinal tissues compared to healthy tissues (Figure S13A–C, Supporting Information). Treatment with hCCL15 activated the human myofibroblast cell line CCD18Co (Figure S13D–F, Supporting Information). These findings suggest that MDSC‐derived mCCL6 and hCCL15 contribute to the profibrotic phenotype of intestinal fibroblasts, thereby promoting the progression of intestinal fibrosis.

### 2.6 The Blockade of the CCL6‐CCR1 Pathway Alleviated Intestinal Fibrosis Symptoms in TNBS‐Treated Mice

**Figure 6:**
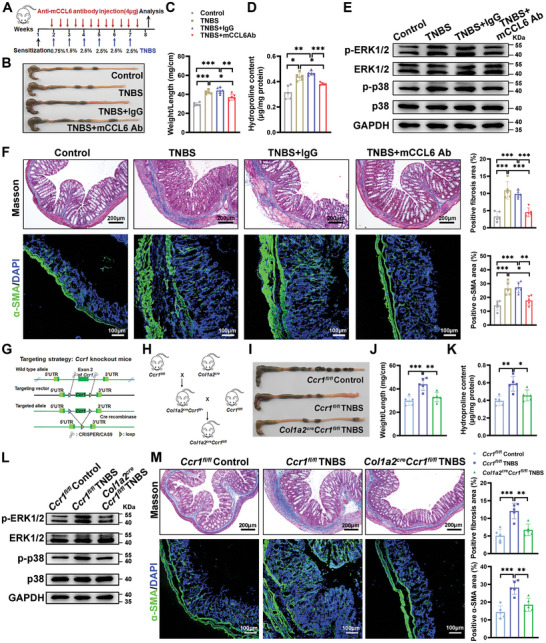
Inhibiting CCL6‐CCR1 pathways mitigated intestinal fibrosis in TNBS‐treated mice. A) The experimental design is depicted for evaluating the therapeutic effects of a mCCL6 antibody on TNBS‐induced intestinal fibrosis. Colonic assessments included B) macroscopic observation, C) determination of the weight‐to‐length ratio, D) hydroxyproline content, and E) Western blotting analysis for p‐ERK1/2 and p‐P38. F) Histological analyses were performed using Masson's trichrome staining and immunofluorescence staining for α‐SMA (Green) with DAPI (Blue) for nuclear staining. *n* = 5 mice per group. G,H) Schematic representation of the strategy for generating fibroblast‐specific *Ccr1* knockout mice. Further colonic assessments included I) macroscopic observation, J) calculation of weight‐to‐length ratio, and K) hydroxyproline content. L) Western blotting was used to evaluate p‐ERK1/2 and p‐P38 levels in colonic tissues of *Ccr1* conditional knockout mice. M) Histological analyses included Masson's trichrome staining and immunofluorescence staining for α‐SMA. *n* = 5 mice per group. F,M) Five fields per tissue section were analyzed, with data points representing the mean of these fields. Representative images are presented. Scale bars are included in (F,M) for reference. The ImageJ software was utilized to quantify the percentages of Masson's trichrome and α‐SMA positive areas. Data are presented as means ± SD. Statistical analyses were performed using Brown‐Forsythe and Welch ANOVA with Tamhane's multiple comparisons test for (D) and one‐way ANOVA with Bonferroni's multiple comparison test for other panels. *p < 0.05, **p < 0.01 and ***p < 0.001.

To investigate the role of mCCL6 in intestinal fibrosis, we treated TNBS‐induced mice with a neutralizing antibody against mCCL6 (**Figure** **6**A). This intervention reduced colon diameter, weight‐to‐length ratio, and hydroxyproline levels, and decreased ERK1/2 and p38 activation in the colon compared to controls (Figure 6B–E). Histological and qPCR analyses further confirmed less tissue damage and lower expression of fibrotic markers in mCCL6 antibody‐treated mice (Figure 6F; Figure S14, Supporting Information). Furthermore, we utilized *Col1a2^cre^Ccr1^fl/fl^* mice, which lack CCR1 specifically in fibroblasts (Figure 6G,H; Figure S15A, Supporting Information), to ascertain the contribution of CCR1 in fibroblast‐mediated fibrogenesis. Deletion of *Ccr1* in fibroblasts using *Col1a2^cre^Ccr1^fl/fl^* mice also ameliorated colonic fibrosis, as evidenced by improved morphological parameters and reduced phosphorylation of ERK1/2 and p38 (Figure 6I–L). Histological and qPCR analyses confirmed a significant reduction in fibrotic tissue damage and lower expression of fibrotic markers in the colon tissues of *Col1a2^cre^Ccr1^fl/fl^* TNBS‐treated mice (Figure 6M; Figure S15B,C, Supporting Information). Consistent with these results, BX471, a CCR1 antagonist, also mitigated colon fibrosis in mice (Figure S16, Supporting Information). These findings underscore the therapeutic potential of targeting the CCL6‐CCR1 signaling pathway for the treatment of intestinal fibrosis.

### 2.7 CCR2 and CXCR2 Ligands Mediated MDSCs Recruitment in Intestinal Fibrosis

**Figure 7:**
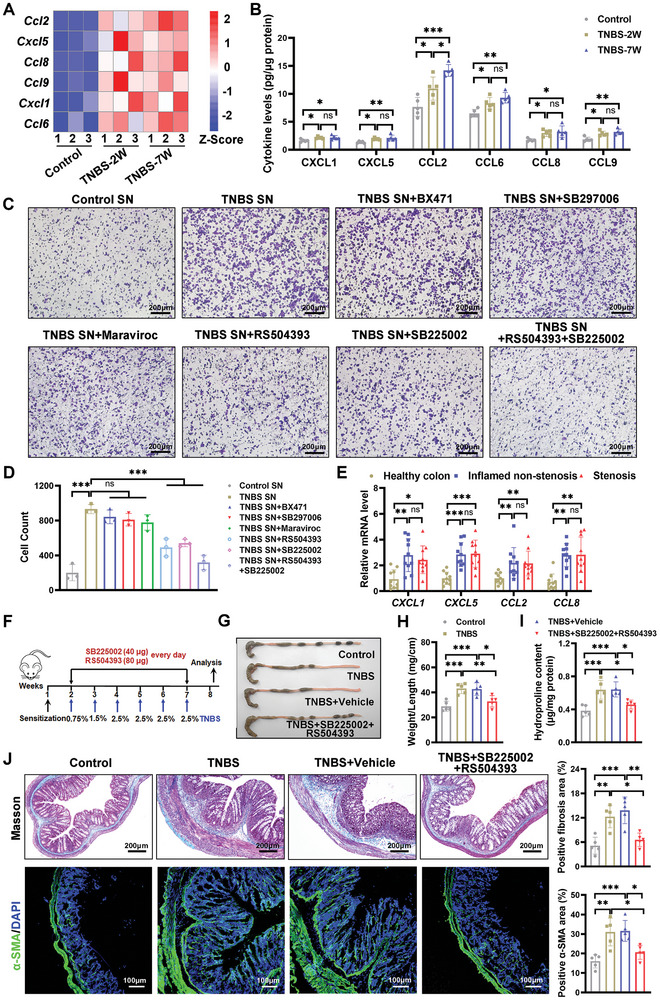
CCR2 and CXCR2 ligands promoted MDSC infiltration into the colonic lamina propria in TNBS‐induced intestinal fibrosis in mice. A) Heatmaps show increased chemokine levels in TNBS‐treated colon tissues at 2 and 7 weeks compared with normal tissues (*p* < 0.05, and |log2FC|>1.0). *n* = 3 mice per group. B) ELISA measurements of CXCL1, CXCL5, CCL2, CCL6, CCL8, and CCL9 proteins in colon tissues from control and TNBS‐induced fibrotic mice at indicated time points. *n* = 5 mice per group. C,D) Bone marrow‐derived MDSCs were pretreated with receptor antagonists (BX471, 20 µm; RS504393, 200 nm; SB297006, 1 µm; Maraviroc, 10 µm; and SB225002, 400 nm) for 1 h before transwell migration assays with colonic tissue supernatants from both control and TNBS‐treated fibrotic mice. Antagonists were present throughout the 12 h chemotaxis assay. Migrated cells were stained with crystal violet and counted. Scale bars represent the image reference. Experiments were repeated three times with similar results. *n* = 3 independent samples. Representative images from five random fields per sample are shown. E) qRT‐PCR analysis of *CXCL1*, *CXCL5*, *CCL2* and *CCL8* in colon tissue from different CD patient regions. *n* = 10 patients. F) Schematic of the therapeutic approach combining SB225002 and RS504393 in a TNBS‐induced fibrosis mouse model. Colonic assessments included G) macroscopic evaluation, H) weight‐to‐length ratio calculation, and I) hydroxyproline content, and J) histological evaluations with Masson's trichrome and immunofluorescence staining for α‐SMA (Green), and DAPI (Blue). Scale bars are included. *n* = 5 mice per group. J) Five fields per tissue section were analyzed, with data points representing the field means. Representative images are presented. ImageJ software quantified the average percentages of Masson's trichrome and α‐SMA positive areas. Data are presented as means ± SD. Statistical analyses were performed using Kruskal‐Wallis test with Dunnett's multiple comparisons test for (E) (*CCL2*, *CCL8*), and one‐way ANOVA with Bonferroni's multiple comparison test for other panels. *p < 0.05, **p < 0.01 and ***p < 0.001, ns, no significant change.

To investigate the mechanisms governing MDSC infiltration into the lamina propria during the development of intestinal fibrosis, we profiled chemokine expression at various stages of a murine intestinal fibrosis model using a PCR array (Figure S17, Supporting Information). Noting the early increase in MDSCs during fibrosis, we focused on chemokines that were consistently upregulated throughout the disease course. The heatmap analysis indicated significant upregulation of *Cxcl1*, *Cxcl5*, *Ccl2*, *Ccl6*, *Ccl8*, and *Ccl9* at both early and late stages of fibrosis in the mouse model (**Figure** **7**A). These trends were validated by ELISA, which confirmed corresponding increases in protein levels (Figure 7B).

Literature indicates that key reports for the chemokines involved are CXCR2 for CXCL1/CXCL5, CCR2 and CCR5 for CCL2, CCR1 for CCL6/CCL9, and CCR1, CCR2, CCR3, and CCR5 for CCL8.^[^ ^28^, ^29^ ^]^ Utilizing a transwell assay, we treated MDSCs with antagonists targeting these receptors: BX471 for CCR1, RS504393 for CCR2, SB297006 for CCR3, Maraviroc for CCR5, and SB225002 for CXCR2. MDSC migration was enhanced by supernatants from TNBS‐treated tissues compared to healthy controls (Figure 7C,D). Blocking CCR2 or CXCR2 significantly inhibited MDSC migration, while individual blockage of CCR1, CCR3, or CCR5 had minor effects. The combined blockade of CCR2 and CXCR2 nearly inhibited MDSC migration by 70% towards supernatants from TNBS‐treated mice (Figure 7C,D). Correspondingly, MDSCs in TNBS‐induced fibrotic mice expressed higher levels of CXCR2 and CCR2 (Figure S18, Supporting Information). qRT‐PCR confirmed increased *CCL2*, *CCL8*, *CXCL1*, and *CXCL5* levels in CD patients’ tissues, highlighting the role of CCR2 and CXCR2 ligands in MDSC recruitment during intestinal fibrosis (Figure 7E). Treatment with RS504393 and SB225002 in a murine model of intestinal fibrosis significantly decreased the colonic MDSC population. This reduction coincided with a substantial decrease in colonic collagen content and a concurrent amelioration of fibrosis symptoms (Figure 7F–J; Figure S19, Supporting Information). In summary, our data suggest that targeting the CCL2/CCL8‐CCR2 and CXCL1/CXCL5‐CXCR2 axes could be a therapeutic strategy for intestinal fibrosis by inhibiting MDSC recruitment.

## 3 Discussion

This study unravels the pivotal role of MDSCs in intestinal fibrosis, particularly in the context of CD. Specifically, we demonstrate that chemokine ligands for CCR2 and CXCR2 orchestrate the sustained recruitment of MDSCs during the fibrosis process in the intestine. Subsequently, MDSCs‐derived mCCL6 stimulates fibroblast activation, thereby promoting the proliferation and deposition of the extracellular matrix, which are hallmarks of fibrosis. The intricate interplay between specialized immune cells, which are predominantly present during chronic inflammation, and effector cells such as fibroblasts, is identified as a key driver of intestinal fibrosis. This understanding paves the way for innovative therapeutic interventions that target these cellular interactions to modulate the fibrotic response.

Despite the established association between inflammatory responses and the progression of intestinal fibrosis, the dynamics of immune cell populations within stenotic intestinal tissues in CD patients have not been fully characterized. Our flow cytometry analysis has identified a consistent increase in MDSCs during the progression of intestinal fibrosis across species, a finding that distinguishes MDSCs from other immune cell populations. The consistent and significant association has prompted us to investigate the role of MDSCs in greater detail. These cells are known to accumulate at site of chronic inflammation or cancer rather than differentiating into mature myeloid cells. For example, various mouse models of colitis, including those induced by dextran sulfate sodium (DSS) or TNBS, as well as IL‐10 knockout mice, have exhibited a significant increase in MDSCs and their subtypes.^[^ ^30^, ^31^ ^]^ Additionally, elevated levels of total MDSCs have been documented in the peripheral blood of CD patients.^[^ ^32^, ^33^ ^]^ Notably, a similar accumulation of MDSCs has been observed in fibrotic tissues of other organs, such as the lungs, kidneys, and heart, underscoring their crucial role in a range of fibrotic conditions.^[^ ^20^, ^34^, ^35^, ^36^ ^]^ Current experiments modulating MDSC ratios in TNBS‐induced models suggest that these cells actively promote the fibrotic process. Based on these findings and previous reports, we propose that the recruitment of MDSCs by mucosal inflammation is a key factor in driving the progression of intestinal fibrosis, rather than being merely an epiphenomenon of the stenotic changes.

The role of MDSCs in fibrosis varies across different organ systems, as highlighted by recent research. In the context of liver and renal fibrosis, MDSCs have been reported to exert anti‐fibrotic effects, potentially through the secretion of IL‐10, which can inhibit fibroblast activation.^[^ ^20^, ^37^ ^]^ However, in pulmonary and myocardial fibrosis, MDSCs are implicated in promoting fibrosis through activation of fibroblasts, with S100A8/A9 and TGF‐β1 playing mediating roles.^[^ ^19^, ^34^ ^]^ Our study did not identify significant changes in factors known to promote or inhibit fibrosis within MDSCs in intestinal fibrotic tissue. Instead, we found mCCL6 to be the most prominently elevated cytokine in intestinal MDSCs from TNBS‐treated mice. In vitro co‐culture experiments confirmed that mCCL6 secreted by intestinal MDSCs directly activates fibroblasts, resulting in enhanced extracellular matrix proliferation. These discrepancies may be due to the plastic and heterogeneous nature of MDSCs, which are influenced by the microenvironment of lesion.^[^ ^38^ ^]^ This highlights the necessity for organ‐specific functional and mechanistic studies on MDSCs in various fibrotic diseases.

mCCL6, primarily produced by intestinal epithelial cells, has antibacterial functions in healthy mice. However, in colitis‐afflicted mice, its elevation enhances the migration of dendritic cells and macrophages, exacerbating colonic inflammation.^[^ ^39^, ^40^ ^]^ We observed substantially higher mCCL6 expression in MDSCs compared to other cellular populations in intestinal fibrosis mouse models. Moreover, both intestinal fibroblasts and immune cells exhibited significantly increased expression levels of mCCL6 receptor, CCR1. Interventions targeting mCCL6 or CCR1 have been shown to alleviate fibrotic symptoms by blocking immune cell infiltration.^[^ ^41^, ^42^ ^]^ Our study extends these findings by revealing the critical role of CCL6‐CCR1 signaling in directly activating intestinal fibroblasts and promoting extracellular matrix accumulation, as demonstrated through antibody neutralization, antagonist treatment or conditional fibroblast‐specific *Ccr1* knockout mice experiments. The human homolog of mCCL6, hCCL15, was also found to effectively activate human fibroblasts, thereby significantly increasing collagen expression. Further mechanistic investigation revealed that mCCL6 binds to CCR1 and activates downstream MAPK signaling pathways, including ERK and p38, both of which are known as key drivers of fibrosis.^[^ ^24^, ^26^, ^27^ ^]^ This explains the potent activation of intestinal fibroblasts by MDSC‐derived mCCL6/hCCL15 and the acceleration of intestinal fibrosis progression.

Through chemokine PCR array and transwell system experiments, we identified CCR2 ligands (CCL2 and CCL8) and CXCR2 ligands (CXCL1 and CXCL5) as critical mediators in the recruitment of MDSCs during intestinal fibrosis. These chemokines were significantly elevated in fibrotic tissues of CD patients with intestinal strictures in our study. The expression patterns of these chemokines were consistent with previous findings in experimental colitis models and CD patients.^[^ ^43^, ^44^ ^]^ These chemokines play pivotal roles in the infiltration of neutrophils and monocytes, thereby contributing to tissue damage.^[^ ^45^, ^46^, ^47^, ^48^ ^]^ Our current research identifies a novel role for CCR2 and CXCR2 ligands in the recruitment of MDSCs during intestinal fibrosis. Antagonist treatments targeting these pathways were found to reduce MDSCs levels in intestinal tissues and mitigate fibrosis symptoms, suggesting that strategies aimed at inhibiting CXCR2‐ and CCR2‐mediated MDSCs recruitment could offer a promising therapeutic approach for the treatment of intestinal fibrosis.

Several aspects of this study warrant further investigation. Notably, the blockade of MDSC‐derived mCCL6 did not fully restore the activation markers of intestinal fibroblasts or the histopathological features of fibrotic intestinal tissue. This partial response may be attributed to the multifactorial nature of intestinal fibrosis. In the TNBS model mice, MDSCs secrete a range of cytokines and chemokines beyond CCL6. Our transcriptome sequencing revealed elevated expression of additional factors such as CCL22, CCL17, CCL4, CCL9, CCL3 and TNFSF12, which, while not yet implicated in intestinal fibrosis, have been linked to fibrosis in other organs.^[^ ^49^, ^50^, ^51^, ^52^ ^]^ Moreover, MDSCs were the primary focus of this study due to the consistent and significant increase in MDSCs across all stages of intestinal fibrosis. Other immune cell populations, despite not showing a significant overall increase, may undergo shifts in their subpopulations or activation states, contributing to the fibrosis process. For example, Th17 and Th2 cells have been implicated in intestinal fibrosis through the production of IL‐17A, Amphiregulin, and IL‐13.^[^ ^27^, ^53^, ^54^ ^]^ Additionally, we recognize that our targeted blockade of CXCR2 and CCR2 axes did not completely inhibit MDSC chemotaxis, suggesting that other, less significantly changed chemokines may also contribute to MDSC recruitment. Future research will focus on deciphering the complex crosstalk among various cell populations and their secreted cytokines to inform the development of multi‐targeted therapeutic strategies.

The study reveals the previously uncharacterized pro‐fibrotic role of MDSCs in the pathogenesis of intestinal fibrosis. Specifically, we have elucidated the underlying mechanistic pathways, highlighting two key aspects: the recruitment of MDSCs by ligands of CXCR2 and CCR2, and the promotion of intestinal fibrosis through activating fibroblasts by MDSCs‐derived mCCL6/hCCL15. These findings not only offer novel insights into the etiology of intestinal fibrosis but also enhance our comprehensive understanding of the multifaceted role of MDSCs in this pathological process.

## 4 Experimental Section

### Human Samples Collection

Full thickness, freshly resected bowel specimens were obtained from CD patients with intestinal stenosis who underwent surgical resection for symptomatic obstruction at Jinling Hospital (Nanjing, China). The study was conducted with ethical approval from the Medical Ethics Committee of Jinling Hospital (approval number 2022DZKY‐048‐01), and written informed consent was obtained from all participants. Clinical characteristics of the patients are detailed in Table S1 (Supporting Information). Intestinal tissues from these CD patients were classified into three segments: healthy intestine (non‐inflammatory and non‐strictured), inflamed non‐stenosis (inflammatory but non‐strictured), and stenosis tissue (inflammatory and strictured) according to a previous study.^[^ ^55^ ^]^ The histology of all specimens was subject to review by an expert pathologist. Tissues were used for histopathologic analysis (H&E, Masson's trichrome, immunohistochemistry, and immunofluorescence), flow cytometry, qRT‐PCR, and Western blotting. Methodological details are provided in the Supporting Information.

### Mice and Induction of Intestinal Fibrosis

Female BALB/c mice (6–8 weeks old) and male C57BL/6JNifdc mice (4 weeks old) were obtained from Beijing Vital River Laboratory Animal Technology Co., Ltd. (Beijing, China). To generate fibroblast‐specific *Ccr1* knockout mice (*Col1a2* ^cre^ *Ccr1^fl/fl^*), *Col1a2*‐2A‐Cre mice (NM‐KI‐215043, Shanghai Model Organisms Center Inc., Shanghai, China) were mated with *Ccr1*‐Flox mice (NM‐CKO‐234050, Shanghai Model Organisms Center Inc.). Then, *Col1a2* ^cre^; *Ccr1^fl/+^* males were mated with *Ccr1^fl/fl^ females to produce Col1a2* ^cre^ *Ccr1^fl/fl^* mice. Tail PCR genotyping methods for mice are shown in the Supporting Information. Mice were housed under specific‐pathogen‐free (SPF) conditions with controlled humidity (50% ± 10%) and temperature (23 °C ± 2 °C) on a 12 h light‐dark cycle. The Ethics Committee for Animal Studies of Nanjing University provided approval for all animal experimental procedures (approval number 2110001‐1).

To induce intestinal fibrosis, the back skin of BALB/c mice and *Col1a2* ^cre^ *Ccr1^fl/fl^* mice, measuring 1.5 × 1.5 cm, was shaved. Mice were then pre‐sensitized with 100 µL solution of a 1% (w/v) solution of TNBS (P2297, Sigma, St Louis, MO, USA) in a vehicle of acetone/olive oil (4:1). Thereafter, mice were intrarectally treated with TNBS in 50% ethanol at gradually increasing concentrations (0.75% once, 1.5% once, and 2.5% four times) weekly for six weeks, following a protocol from a previous study.^[^ ^56^ ^]^ Control mice received an equivalent volume of 50% ethanol. At indicated time points, colonic tissues were harvested for macroscopic observation, histopathological assessment, primary cell isolation, hydroxyproline quantification, qRT‐PCR, PCR array analysis, Western blotting, flow cytometry, and ELISA.

### Primary Cell Isolation and Cell Culture

The human intestinal myofibroblast cell line CCD18Co was sourced from ATCC (Rockville, Maryland, USA) and the murine colon adenocarcinoma cell line MC38 was obtained from the Cell Bank of the Chinese Academy of Sciences (Shanghai, China). Primary mouse colonic epithelial cells, fibroblasts and lamina propria mononuclear cells (LPMCs) from mouse and CD patients were isolated by following established protocols.^[^ ^57^, ^58^, ^59^ ^]^ Mouse colonic MDSCs were further isolated from LPMCs using magnetic bead separation, and MDSCs for in vivo adoptive transfer were induced from the bone marrow of C57BL/6JNifdc mice. Detailed methods are described in the Supporting Information. Cells were cultured in appropriate media: CCD18Co in Eagle's minimum essential medium (EMEM) (30‐2003, ATCC) with 10% fetal bovine serum (FBS) (Life Technologies, Grand Island, NY, USA), MC38 and primary MDSCs in Dulbecco's modified Eagle medium (DMEM) (319‐005‐CL, WISENT, Nanjing, China) with 10% FBS, and primary mouse intestinal fibroblasts in DMEM containing 10% FBS, 1% penicillin/streptomycin (BC‐CE‐007, SenBeiJia Biological Technology Co., Ltd., Nanjing, China), and 0.5 µg mL^−1^ Fungizone (V900919, Sigma). All human cell lines were verified by STR DNA profiling, and PCR assays were performed to detect mycoplasma contamination.

### Cell Treatments and Assays

To assess the effect of mCCL6 and hCCL15 on the activation and collagen synthesis of colon fibroblasts, 5 × 10^5^ colon fibroblasts from healthy mice or the human myofibroblast cell line CCD18Cos were seeded in six‐well plates and serum‐starved for 6 h. The cells then treated with 25 ng mL^−1^ mCCL6 (250‐06‐10, PeproTech, Rocky Hill, NJ, USA) or 20 ng mL^−1^ hCCL15 (300‐43‐25, PeproTech) for 24 h, respectively. The expression of α‐SMA, Col I, Col III and Col VI in the treated fibroblasts was examined using qRT‐PCR, immunofluorescent staining and Western Blotting. To examine the impact of mCCL6 on fibroblast signaling pathways, mouse colon fibroblasts were serum‐starved for 6 h prior to treatment with 25 ng mL^−1^ mCCL6 for 30 min. Phosphorylation levels of ERK1/2 and p38 were analyzed by Western blotting.

A co‐culture model was established to investigate the mechanism of MDSCs in intestine fibrosis. Primary colonic fibroblasts (2×10^5^) from healthy mice were seeded in 2 mL of DMEM in the lower chamber of a transwell system (0.4 µm, #3450, Corning, NYC, NY, USA), and primary colonic MDSCs from healthy or TNBS‐treated mice were added to the upper chamber in 1 mL of DMEM at varying ratios. The optimal co‐culture ratio of fibroblasts to MDSCs (1:10) was determined by preliminary experiments measuring Acta2 mRNA levels in fibroblasts using qRT‐PCR. In some experiments, mCCL6‐neutralizing antibodies (40 ng mL^−1^, MAB487, R&D Systems, Emeryville, CA, USA) or the CCR1 antagonist BX471 (20 µm, HY‐12080, MCE, Jersey, NJ, USA) were applied to MDSCs or fibroblasts 6 h before coculture to block the CCL6‐CCR1 axis and were present throughout to inhibit fibroblast activation. The levels of p‐ERK1/2 and p‐p38 (after 30 min of coculture), and α‐SMA, Col I, Col III and Col VI (after 24 h of coculture) in colonic fibroblasts were analyzed using qRT‐PCR, immunofluorescent staining or Western Blotting.

To explore MDSCs’ role in epithelial‐mesenchymal transition (EMT), MC38 cells were cocultured with colonic MDSCs from TNBS‐treated mice using the transwell system at a 1:10 ratio for 24 h. The expression of EMT markers (*Cdh1*, *Acta2*, *Snail*, *Fsp1*, and *Vimentin*) in MC38 cells were examined by qRT‐PCR.

### Chemotaxis Assays

To determine the chemokines mediating MDSC recruitment in fibrosis, 200 µL of complete medium containing 3×10⁵ bone marrow‐derived MDSCs were placed in the upper chamber of a transwell insert (24‐well, 8 µm pore size; Corning). The lower chamber was filled with 600 µL of supernatants from colon tissue of normal or fibrotic mice. Supernatants were prepared as previously described,^[^ ^60^ ^]^ where minced colon tissue fragments (100 mg) from control and TNBS‐treated mice, pre‐treated with DMEM/F12 medium containing 5% FBS, penicillin (100 U mL^−1^), streptomycin (0.1 mg mL^−1^), and gentamycin (20 µg mL^−1^) for 2 h, were then incubated in 1 mL of DMEM/F12 medium containing 5% FBS for 12 h. The mixture was filtered through a cell strainer and centrifuged at 12000 × *g* for 5 min. The resulting supernatant was collected for the chemotaxis assay. In selected experiments, MDSCs were pre‐incubated with antagonists for CCR1 (BX471, 20 µm, HY‐12080, MCE), CCR2 (RS504393, 200 nm, HY‐15418, MCE), CCR3 (SB297006, 1 µm, HY‐103361, MCE), CCR5 (Maraviroc, 10 µm, S2003, Selleck, Houston, Texas, USA), or CXCR2 (SB225002, 400 nm, S7651, Selleck) for 1 h before exposure to colon tissue supernatants. Antagonists remained present throughout the 12 h incubation in the transwell system. Non‐migrating cells were gently removed, and migrating cells in the lower chamber were fixed with 4% paraformaldehyde and stained with 0.1% crystal violet (RS3200, G‐CLONE, Beijing, China). Cell counts were performed in five random fields at 100× magnification using a Nikon microscope (C2+, Nikon, Tokyo, Japan).

### Experiments in TNBS‐Induced Intestinal Fibrosis Model

To investigate the role of MDSCs in intestinal fibrogenesis, BALB/c mice were administered intraperitoneal injections of anti‐Gr‐1 antibody (BP0075, BioXcell, West Lebanon, NH, USA) at 100 µg per mouse thrice weekly, or intravenously injected with 5×10^6^ bone marrow‐induced MDSCs twice weekly during the TNBS‐induced model establishment.

To evaluate the impact of MDSC‐derived mCCL6 on intestinal fibrosis, mice were intraperitoneally treated with a mCCL6 neutralizing antibody (4 µg per mouse, MAB487, R&D Systems) or an isotype IgG2B control (MAB0061, R&D Systems) one day before and three days after the intracolonic administration of TNBS. Additionally, to investigate the effect of CCR1 blockade, the CCR1 antagonist BX471 or vehicle solution (10% DMSO+40% PEG300+5% Tween‐80+45% saline) was administered subcutaneously at 0.4 mg per mouse daily throughout the 6 week fibrosis model. The efficacy and specificity of the anti‐Gr‐1 and anti‐mCCL6 antibodies were confirmed by the suppliers and the preliminary experiments.

To investigate the impact of blocking CXCR2‐ and CCR2‐ligand‐mediated MDSC recruitment on intestinal fibrosis, mice were treated daily with a CXCR2 antagonist (SB225002, 40 µg per mouse, intraperitoneally) and a CCR2 antagonist (RS504393, 80 µg per mouse, orally) throughout the 6 week fibrosis model. Post‐treatment, colon tissues from mice subjected to various treatments were collected for macroscopic observation, histopathological examination, primary cell isolation, hydroxyproline assay, qRT‐PCR, PCR array, Western blotting, flow cytometry, and ELISA. Detailed methods are provided in the Supporting Information, with antibody and gene primer information in Tables S2 and S3 (Supporting Information). The primers were synthesized by Sangon Biotech (Shanghai) Co., Ltd.

### Statistical Analysis

Data normality was initially assessed using the Shapiro‐Wilk's test in GraphPad Prism 8 software (GraphPad Software Inc. La Jolla, CA, USA). Once normal distribution and homogeneity of variance were confirmed, the appropriate tests were selected: unpaired student's t‐test (two tailed) for two‐group comparisons, one‐way ANOVA with Bonferroni's post hoc test for multiple group comparisons, two‐way ANOVA with Bonferroni's post hoc test for interactions, or Pearson's correlation analysis for assessing relationships between variables. For data that did not meet the normality criterion, nonparametric tests were applied, including the Mann‐Whitney U test (two‐tailed), or Kruskal‐Wallis one‐way ANOVA analysis followed by Dunn's multiple comparisons test. In cases of normal distribution with unequal variances, the t test with Welch's correction (two tailed), or Brown‐Forsythe and Welch ANOVA with Tamhane's multiple comparisons test were used. Data were presented as mean ± standard deviation (Mean ± SD). Statistical significance was set as *p* < 0.05, and “ns” meant no significant difference.

## Conflict of Interest

The authors declare no conflict of interest.

## Author Contributions

Z.H., J.C., and J.Z. designed the experiments, and supervised all aspects of study. X.C., P.S., X.W., J.J., and J.C. carried out the experimental work. X.C., X.W., and P.S. analyzed the data. Z.H., J.C., and J.Z. contributed reagents/materials/analysis tools. Y.L. and W.Z. supported the clinical sample analysis and supervised the study. X.C. prepared the manuscript. Z.H. and J.C. revised the manuscript.

## Supporting information



Supporting Information

## Data Availability

The data that support the findings of this study are available in the Supporting Information of this article or from the corresponding author upon reasonable request.
