# Supplementary material for: Myeloid‐Derived Suppressor Cell Accumulation Drives Intestinal Fibrosis through mCCL6/hCCL15 Chemokine‐Mediated Fibroblast Activation
Source: Adv Sci (Weinh). 2024 Dec 31;12(8):2411711. doi: 10.1002/advs.202411711 (PMC11848553; doi:10.1002/advs.202411711)
Supplement: Supplementary file 1 — Supporting Information [file ADVS-12-2411711-s001.docx]

Supporting Information

**Myeloid-Derived Suppressor Cell Accumulation Drives Intestinal Fibrosis through mCCL6/hCCL15 Chemokine-Mediated Fibroblast Activation**

*Xiaohui Cheng^1^, Pingwen Shao^1^, XinTong Wang^1^, Juan Jiang^1^, Jiahui Chen^1^, Jie Zhu^1^, Weiming Zhu^3^, Yi Li^3*^, Junfeng Zhang^1*^, Jiangning Chen^1,2*^, Zhen Huang^1,4*^*

^1^State Key Laboratory of Pharmaceutical Biotechnology, School of Life Sciences, Nanjing University, Nanjing, Jiangsu, 210023, China

^2^State Key Laboratory of Analytical Chemistry for Life Sciences, Nanjing University, Nanjing, Jiangsu, 210023, China

^3^Department of General Surgery, Jinling Hospital, School of Medicine, Nanjing University, Nanjing, Jiangsu, 210002, China

^4^NJU Xishan Institute of Applied Biotechnology, Xishan District, Wuxi, Jiangsu, 214101, China

***Correspondence:** Zhen Huang ([zhenhuang@nju.edu.cn](mailto:zhenhuang@nju.edu.cn)), Jiangning Chen (jnchen[@nju.edu.cn](mailto:zhenhuang@nju.edu.cn)), Junfeng Zhang (jfzhang@nju.edu.cn), or Yi Li (liyi.jlh@hotmail.com).

**Supplementary methods**

*Isolation of primary intestinal epithelial cells, LPMCs, and fibroblasts*: Intestinal tissues from both mice and CD patients were longitudinally sectioned, thoroughly rinsed in ice-cold 1×PBS, and segmented into 1 cm fragments. These fragments underwent a pre-digestion process at 37°C with gentle shaking (200 rpm) in 1×HBSS (14175095, Gibco, Waltham, MA, USA) supplemented with 5 mM EDTA (A610185, Sangon Biotech, Shanghai, China) and 1 mM DTT (A100281, Sangon Biotech) for 20 minutes. Following a 30-second vortex, the pre-digestion fluid was collected, and sequential centrifugation and washes yielded the intestinal epithelial cells.^[1]^

Subsequent to additional PBS washes, the residual intestinal tissues were finely minced to 1 mm pieces prior to digestion in a cocktail of collagenase II (1.5 mg/mL, C6885, Sigma, St Louis, MO, USA), DNase I (0.5 mg/mL, A610099, BBI Life Science Corporation, Shanghai, China), and hyaluronidase (0.3 mg/mL, H4272, Sigma) in DMEM at 37°C with rotation at 200 rpm for 20 minutes. The cell suspension obtained was then strained through a 70 μm cell filter and the lamina propria mononuclear cells (LPMCs) were isolated using Percoll density gradient centrifugation according to a previous report.^[2]^

To obtain primary intestinal fibroblasts, the cell suspension obtained from above mentioned enzyme digestion were cultured with DMEM containing 10% FBS (Life Technologies, Grand Island, NY, USA), 1% penicillin/streptomycin (BC-CE-007, SenBeiJia Biological Technology Co., Ltd., Nanjing, China), and 0.5 µg/mL Fungizone (V900919, Sigma) in a 25 cm^2^ flask. After seeding, non-adherent cells were systematically dislodged by renewing the medium after 1, 2, and 6 hours. The remaining cells were identified as intestinal fibroblasts.

The purity of the intestinal epithelial cells and LPMCs was examined with flow cytometry by staining with Alexa Fluor 488 anti-cytokeratin (PCK) antibody (purity > 90%, 628608, Biolegend, San Diego, CA, USA) or FITC anti-mouse CD45 antibody (purity > 90%, 157214, Biolegend) and used for qRT-PCR. Additionally, LPMCs were used for flow cytometry analysis and primary MDSC isolation. Cells isolated from MDSCs were collected as other LPMCs. Primary intestinal fibroblasts, validated by vimentin immunostaining and morphological criteria, were cultivated under these conditions for passages 3 to 8 for subsequent cell experiments.^[3]^

*MDSCs induction and culture*: For adoptive cell transfer, MDSCs were induced from bone marrow cells extracted from four-week-old male C57BL/6JNifdc mice. In brief, the cells were filtered through a 70 μm cell strainer, and red blood cells were lysed using red blood cell lysis buffer. The isolated cells were cultured in DMEM medium, enriched with 10% FBS, 1% streptomycin/penicillin, along with the addition of mouse recombinant GM-CSF (40 ng/mL, 315-03-20, Peprotech, Rocky Hill, NJ, USA) and IL-6 (40 ng/mL, 216-16-20, Peprotech), for a continuous period of 4 days.^[4]^

For the isolation of primary colonic MDSCs, cell fractions from the colon lamina propria of both control and TNBS-treated mice were stained with an Fc blocker on ice for 15 minutes and subjected to staining with PE-Gr-1 antibody (108408, Biolegend) for 30 minutes. The PE-labeled MDSCs were separated using R-PE IMag Particles-DM magnetic beads (557899, BD Biosciences, San Jose, NJ, USA). Subsequently, sorted cells were lysed for total RNA extraction using an RNA purification kit (NGB-51800, Norgen Biotek Corp, Thorold, Canada). Flow cytometry analysis was performed to assess the purity of both bone-marrow derived MDSCs and primary colonic MDSCs, which were identified as cells expressing both CD11b and Gr-1 markers.

*Mouse Genotyping:* Genomic DNA was extracted from tail biopsies of mice using the Quick Genotyping Assay Kit (Beyotime, Shanghai, China). Primer sequences for genotyping are listed in Table S3 (Supporting Information). For the PCR reaction, 100 ng of genomic DNA and the corresponding primers were mixed with 2× Taq Plus Master Mix II (Dye Plus) (Vazyme, P213-01, Nanjing, China). The PCR protocol consisted of an initial denaturation at 98℃ for 3 minutes, followed by 35 cycles of denaturation at 98℃ for 10 seconds, annealing at 63℃ for 10 seconds, extension at 72°C for 60 seconds, and a final extension at 72℃ for 5 minutes. The PCR products were separated by agarose gel electrophoresis, yielding the following expected band sizes: *Ccr1^fl/fl^* WT: 240 bp, heterozygote: 297 bp and 240 bp, homozygote: 297 bp; *Col1a2^cre^* WT: 366 bp, heterozygote: 366 bp and 440 bp, homozygote: 440 bp.

*Flow cytometry analysis*: To evaluate the composition of various immune cell populations in the intestinal tissue of both CD patients and mice with intestinal fibrosis, single-cell suspensions from the lamina propria were prepared. The procedure involved blocking Fc receptors with anti-CD16/CD32 antibodies (101320, Biolegend), identifying dead cells using either 7-AAD Viability Staining Solution (420403, Biolegend) for surface staining or the Zombie Violet™ Fixable Viability Kit (423113, Biolegend) for intracellular staining, and staining cell surface molecules with corresponding fluorescent antibodies. Permeabilization was performed using Cytofix/Cytoperm (554715, BD Biosciences). Subsequently, cells were stained for antibodies against mCCL6, Foxp3, CD68, or hCCL15. Flow cytometry analysis was conducted using the Invitrogen™ Attune™ NxT system (Thermo Fisher Scientific, Waltham, MA, USA). For t-SNE data analysis, we utilized the concatenate function in FlowJo 10.4 software to integrate each group of samples into a single FCS file. An island plot was then generated using t-SNE, and a gate was manually set to identify various cell populations, which were subsequently overlaid onto the t-SNE plot. Antibodies used for flow cytometry are listed in Table S2 (Supporting Information).

*H&E and Masson's trichrome staining:* Intestinal tissues were harvested, fixed in 4% paraformaldehyde, paraffin-embedded, and serially sectioned. Morphological alterations were assessed using H&E staining, with kits from Beijing Leagene Biotech Co., Ltd. (DH0006, Beijing, China). The thickness of the intestinal tissue layers in patients was measured using ImageJ software. To evaluate fibrosis, sections were stained with modified Masson's trichrome stain following the manufacturer's protocol (TRM-2, ScyTek Laboratories, UT, USA). The extent of fibrosis was quantitatively assessed by calculating the fibrotic area relative to the total field area in selected fields using ImageJ software (Media Cybernetics, Bethesda, MD, USA).

*Immunohistochemical staining:* For immunohistochemistry, tissue sections were deparaffinized, rehydrated, and incubated in a 3% hydrogen peroxide solution for 10 minutes to block endogenous peroxidase activity. Antigen retrieval was achieved by heating the sections in citrate antigen retrieval solution (pH 6.0) (P0083, Beyotime) at 95°C for 20 minutes. Non-specific antibody binding was then blocked by incubating the sections in 5% BSA solution (ST023, Beyotime) for 1 hour. The sections were subsequently incubated with primary antibodies overnight at 4°C, followed by incubation with a corresponding secondary antibody (GK500510A, GeneTech, Shanghai, China) for 1 hour. Staining was performed using a DAB chromogenic substrate kit (AR1027-3, Boster, Wuhan, China) for 5 minutes, and nuclei were counterstained with hematoxylin (DH0001, Leagene) for 60 seconds.

IHC staining images were captured using an Eclipse Ni-E upright microscope (Nikon, Tokyo, Japan) and analyzed using the semiquantitative immunoreactive score (IRS) system via ImageJ software. The data were analyzed in a blinded manner. The intensity of immunostaining was scored from 0 to 3 (0 = negative, 1 = weak, 2 = moderate, 3 = strong), and the percentage of positively stained cells was scored from 1 to 4 (1 = 0–25%, 2 = 26–50%, 3 = 51–75%, 4 = 76–100%). The IRS was calculated by multiplying the intensity score by the percentage score, yielding a total score ranging from 0 to 12 for each sample. Antibodies used for immunopathological staining were listed in Table S2 (Supporting Information).

*Immunofluorescence staining:* Serial tissue slices were placed on adherent slides and subjected to gradient hydration. Antigen retrieval was performed using heat-mediated improved citrate antigen retrieval solution (pH 6.0) (P0083, Beyotime) for 20 minutes at 95°C, following the manufacturer's instructions. To block nonspecific binding, the slices were incubated in 5% BSA solution for 1 hour at room temperature. After overnight incubation with primary antibodies at 4°C and subsequent washing, secondary antibodies were applied in 1% BSA for 1 hour at room temperature in the dark, with DAPI (C1005, Beyotime) used to stain nuclei.

Colonic fibroblasts, cultured on poly-D-lysine-coated glass slides, were fixed in 4% paraformaldehyde (PFA), permeabilized with 0.2% Triton X-100 (ST797, Beyotime), blocked with 5% BSA, and incubated with primary antibodies at 4°C overnight. This was followed by treatment with corresponding secondary antibodies for 1 hour at room temperature, concluding with DAPI nuclear staining. Images were captured using a Zeiss LSM980 confocal microscope and analyzed with ZEN 3.4 software. The antibodies used for immunofluorescence staining are listed in the Table S2 (Supporting Information).

*Western blotting*: Intestinal tissue and cellular proteins were lysed using RIPA lysis buffer (P0013B, Beyotime) supplemented with the protease inhibitor PMSF (ST2573, Beyotime) and phosphatase inhibitors (P1082, Beyotime). Protein concentrations were determined using the bicinchoninic acid (BCA) protein assay kit (PA115, Tiangen Biotech Co., Ltd., Beijing, China). The proteins were separated on a 10% SDS-polyacrylamide gel and transferred onto a polyvinylidene fluoride (PVDF) membrane (Millipore, Billerica, MA, USA). The membranes were blocked with 5% BSA and incubated overnight at 4°C with primary antibodies, followed by incubation with HRP-conjugated secondary antibodies. GAPDH was used as the loading control. Protein bands were visualized using an ECL detection reagent (Thermo Fisher Scientific). The antibodies used for Western blotting are listed in the Table S2 (Supporting Information).

*RNA sequencing*: RNA sequencing was performed on primary colonic MDSCs from both healthy mice and TNBS-treated mice. This process utilized Next Generation Sequencing (NGS) on the Illumina HiSeq-2500 platform, operated in high output mode by Shanghai Bohao Biotechnology Co., Ltd. Library preparation for sequencing was performed using the VAHTS Universal V6 RNA-seq Library Prep Kit. Differential gene expression (DGE) analysis followed, employing the Benjamini-Hochberg method for false discovery rate correction, with a focus on genes exhibiting a log2 fold change greater than 1.0.

*qRT-PCR assay*: RNA was extracted from cultured cells, primary isolated cell fractions, and tissue samples using TRIzol reagent (15596026, Invitrogen, Thermo Fisher Scientific) following standard protocols. The purified RNA was reverse-transcribed into cDNA using the HiScript III RT SuperMix for qPCR (+gDNA wiper) kit (R323-01, Vazyme) according to the manufacturer's instructions. Gene expression analysis was conducted using qRT-PCR with the ChamQ Universal SYBR qPCR Master Mix (High ROX Premixed) (Q341, Vazyme) on an Applied Biosystems StepOnePlus Real-Time PCR System (Thermo Fisher Scientific). The primers used for the qRT-PCR assays are listed in Table S3 (Supporting Information). The expression profiles of chemokine-related genes in the mouse colon were examined using a chemokine and receptor PCR array plate, following the manufacturer's protocol (WC-MRN40033-M, Wcgene Biotech, Shanghai, China). The data were analyzed using Wcgene Biotech software and presented as a heatmap generated by HemI (Heatmap Illustrator, version 1.0, HemI (Heatmap Illustrator, version 1.0, Wuhan, China).

*ELISA Detection:* Intestinal tissue samples from mice were collected and homogenized in cold PBS using a rotor-stator homogenizer with 5 mm beads (two 60-second cycles at 60 Hz; Tissuelyser-24, Jingxin Industrial Development Co., Ltd., Shanghai, China). The homogenates were then centrifuged at 12,000 rpm for 10 minutes at 4°C, and the resulting supernatants were collected. Protein concentrations in the supernatants were measured using the BCA protein assay kit (Tiangen Biotech Co., Ltd.). The levels of specific cytokines—including CCL2 (EK287, Multi Sciences, Hangzhou, China), CCL6 (EK1125, Boster), CCL8 (EK1137, Boster), CCL9 (EK1225, Boster), CXCL1 (EK296, Multi Sciences), and CXCL5 (EK0919, Boster)—were quantified following the manufacturer's protocols. Absorbance was measured at 450 nm using a Varioskan LUX multifunctional microplate reader (Thermo Fisher Scientific)*.*

*Hydroxyproline content assay:* The hydroxyproline content in intestinal tissues was measured using a Hydroxyproline Assay Kit (A030-2-1, JianCheng Bioengineering Institute, Nanjing, China) following the manufacturer's protocol and a previously described method.^[5]^ Briefly, approximately 30 mg of wet intestinal tissue from mice was weighed and transferred into a test tube, followed by the addition of 1 mL of hydrolysate solution. The samples were thoroughly mixed, sealed, and hydrolyzed in a water bath at 95°C for 20 minutes. After hydrolysis, the pH of the lysates was adjusted to 6.0-6.8, and the volume was brought to 10 mL with distilled water. The mixture was then thoroughly mixed again. Subsequently, 3-4 mL of the diluted hydrolysate was treated with activated carbon, mixed, and centrifuged at 3,500 rpm for 10 minutes. A 1 mL aliquot of the clear supernatant was collected for analysis. The collagen content was quantified by reacting oxidized hydroxyproline with dimethylaminobenzaldehyde (DMAB), producing a colorimetric product measurable at 550 nm. Results were expressed as µg of hydroxyproline per mg of protein.

**References**

[1] C. Marchiori, M. Scarpa, A. Kotsafti, S. Morgan, M. Fassan, V. Guzzardo, A. Porzionato, I. Angriman, C. Ruffolo, S. Sut, S. Dall'Acqua, R. Bardini, R. De Caro, C. Castoro, M. Scarpa, I. Castagliuolo, *J Exp Clin Cancer Res*. **2019**, 38, 190.

[2] B. Weigmann, I. Tubbe, D. Seidel, A. Nicolaev, C. Becker, M.F. Neurath, *Nat Protoc*. **2007**, 2, 2307.

[3] D.Q. Shih, L. Zheng, X. Zhang, H. Zhang, Y. Kanazawa, R. Ichikawa, K.L. Wallace, J. Chen, C. Pothoulakis, H.W. Koon, S.R. Targan, *Mucosal Immunol*. **2014**, 7, 1492.

[4] Y. Bu, Q. Liu, Y. Shang, Z. Zhao, H. Sun, F. Chen, Q. Ma, J. Song, L. Cui, E. Sun, Y. Luo, L. Shu, H. Jing, X. Tan, *Int J Biol Macromol*. **2024**, 270, 131949.

[5] J. Liang, Y. Zhang, T. Xie, N. Liu, H. Chen, Y. Geng, A. Kurkciyan, J.M. Mena, B.R. Stripp, D. Jiang, P.W. Noble, *Nat Med*. **2016**, 22, 1285.

**Supplementary figure**


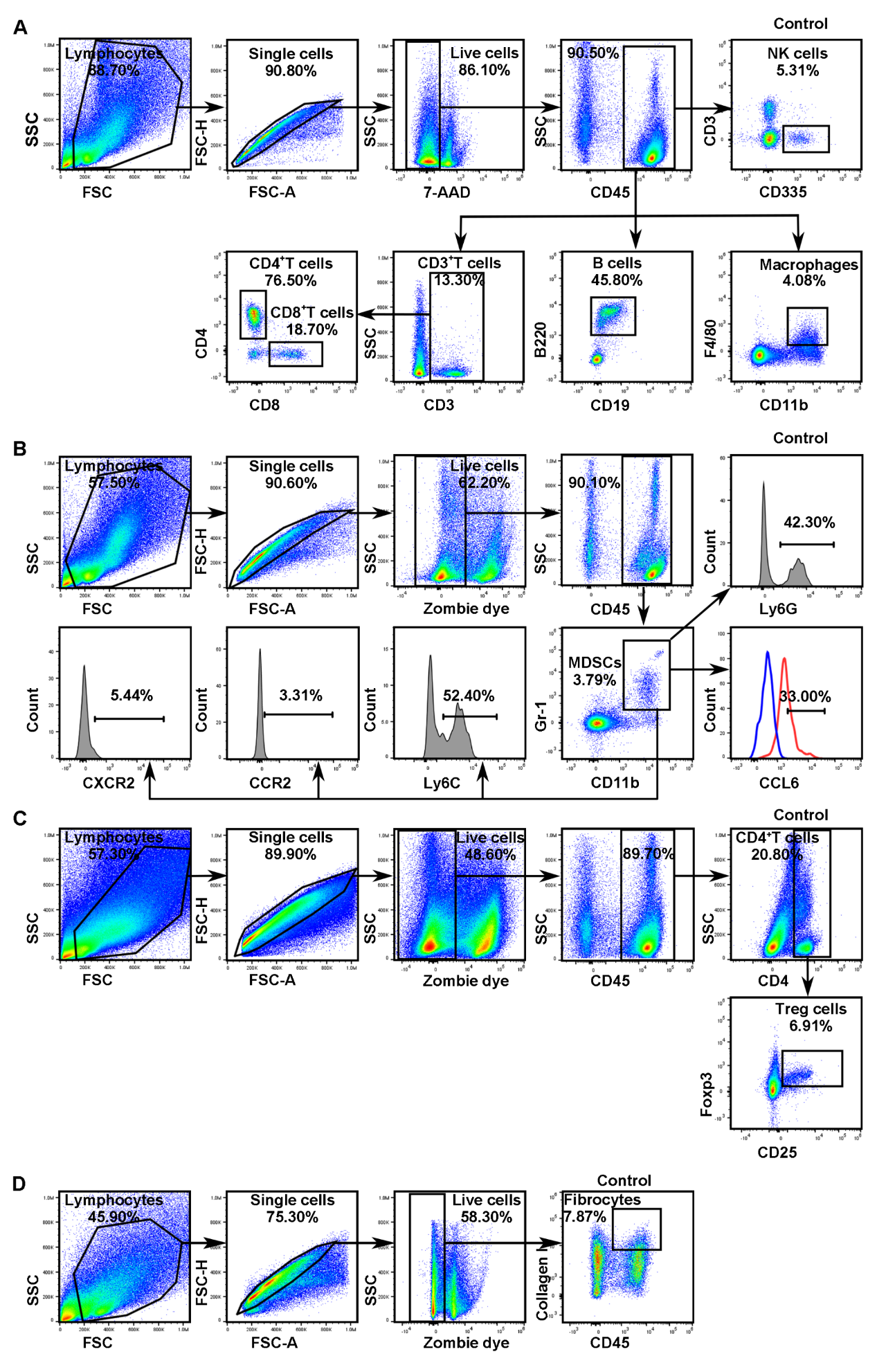


**Figure S1. Flow cytometry gating strategy for colon cell populations in mice.** A) Gating strategies were employed to analyze immune cells, including B cells (CD19^+^B220^+^), CD4^+^ T cells (CD3^+^CD4^+^), CD8^+^ T cells (CD3^+^CD8^+^), macrophages (CD11b^+^F4/80^+^), and NK cells (CD3^-^CD335^+^) within CD45^+^ leukocytes in the colon. B) MDSCs (CD11b^+^Gr-1^+^) and their subpopulations: M-MDSCs (CD11b^+^Gr-1^+^Ly6C^+^) and PMN-MDSCs (CD11b^+^Gr-1^+^Ly6G^+^)) and the expression of CCL6 (CCL6^+^), CCR2 (CCR2^+^), and CXCR2 (CXCR2^+^) in MDSCs among CD45^+^ leukocytes in the colon. C) Tregs (CD4^+^CD25^+^Foxp3^+^) within CD45^+^ leukocytes in the colon. D) Fibrocytes (CD45^+^Collagen Ⅰ^+^) among live cells in the colon.


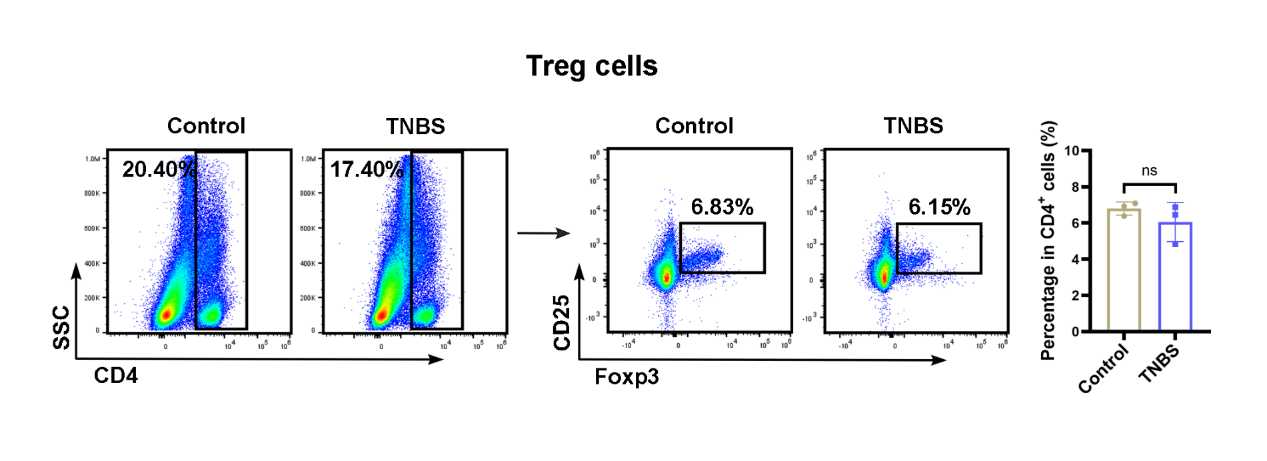


**Figure S2. The proportion of Treg cells (CD4^+^CD25^+^Foxp3^+^) in the colonic lamina propria of control mice and TNBS-induced intestinal fibrosis mice was assessed by flow cytometry.** n = 3 mice per group. Data are represented as mean ± SD. Statistical analysis was performed using unpaired Student’s t test (two tailed). ns, not significant.


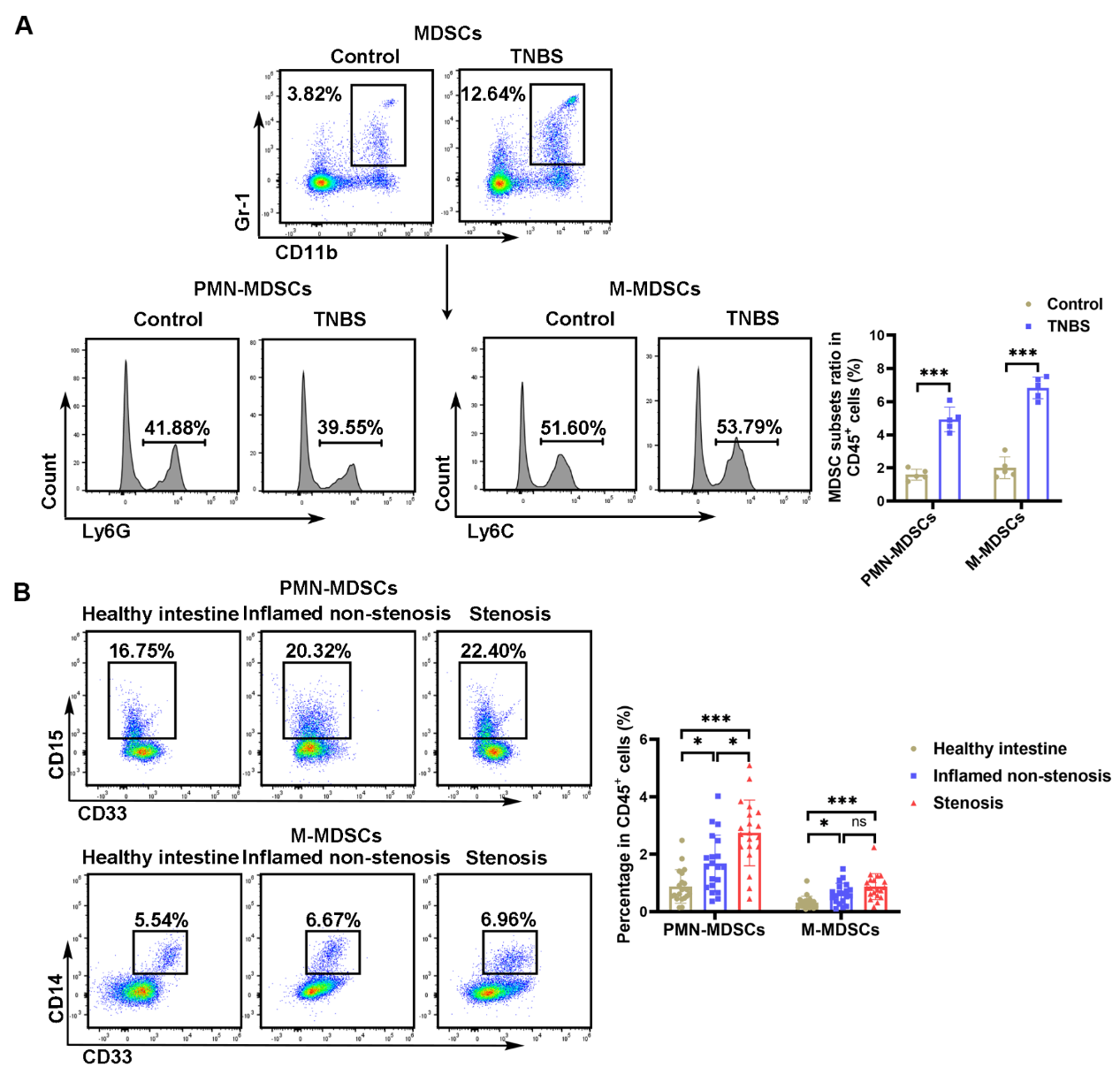


**Figure S3. Analysis of intestinal MDSC subpopulations in the TNBS-induced** **intestinal fibrosis mouse model and in CD patients. A)** Flow cytometry was used to determine the proportions of M-MDSCs and PMN-MDSCs within the colonic lamina propria cells of the mouse model. n = 5 mice per group. **B)** Flow cytometry analysis was conducted to determine the proportions of M-MDSCs (CD33^+^CD11b^+^HLA-DR^-/low^CD14^+^) and PMN-MDSCs (CD33^+^CD11b^+^HLA-DR^-/low^CD15^+^) in healthy intestinal tissue, inflamed non-stenotic intestinal tissue, and inflamed stenotic intestinal tissue from CD patients (n = 20). Data are presented as means ± SD. Statistical analysis for panel A was performed using an unpaired Student’s t test (two tailed), while panel B was analyzed using the Kruskal-Wallis test with Dunn’s multiple comparisons test. **P <* 0.05, and ****P <* 0.001, ns, not significant.


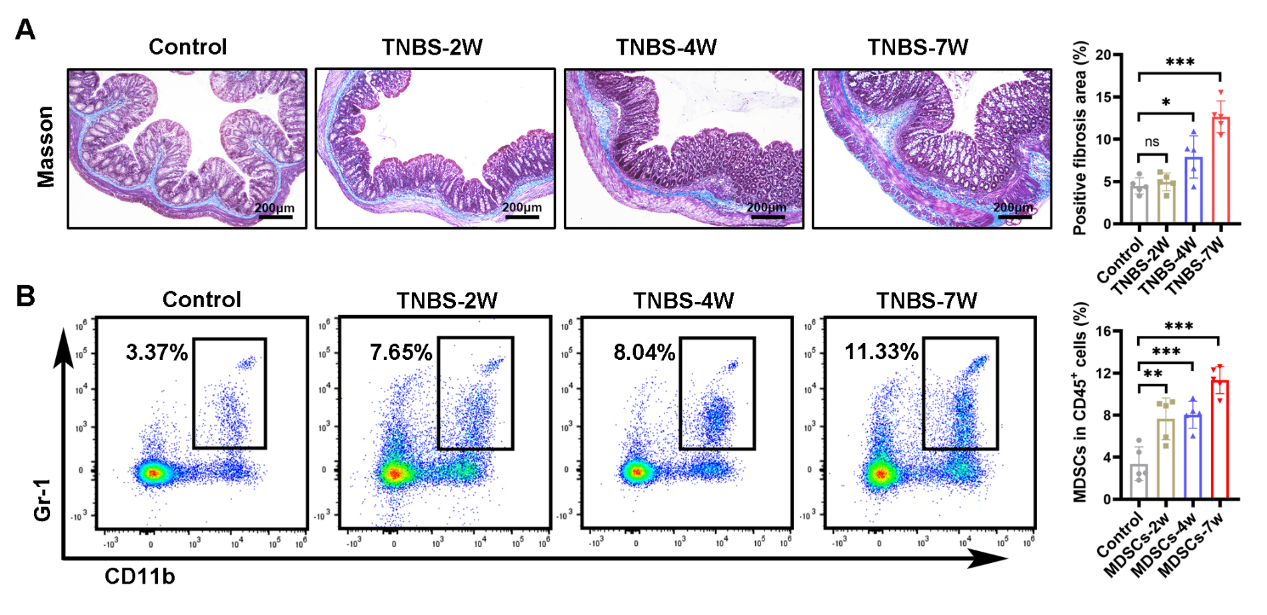


**Figure S4. Evaluation of collagen deposition and MDSC proportions in the colon tissue at different stages of the TNBS-induced intestinal fibrosis model.** A) Masson's trichrome staining was used to visualize collagen deposition in the colon at 2, 4, and 7 weeks post-TNBS treatment. The mean percentage of positively stained areas was quantified using ImageJ software, with scale bars included for reference. n = 5 mice per group. For each mouse, tissue sections were examined, and five fields were captured for quantitative analysis, with data points representing the mean of these fields. Representative images are presented. B) Flow cytometry was conducted to assess MDSC proportions in the colonic lamina propria at indicated time points following TNBS treatment. n = 5 mice per group. Data are presented as means ± SD. Statistical analysis was performed using one-way ANOVA with Bonferroni’s post hoc test for multiple comparisons. **P* < 0.05, ***P* < 0.01 and ****P* < 0.001, ns, not significant.


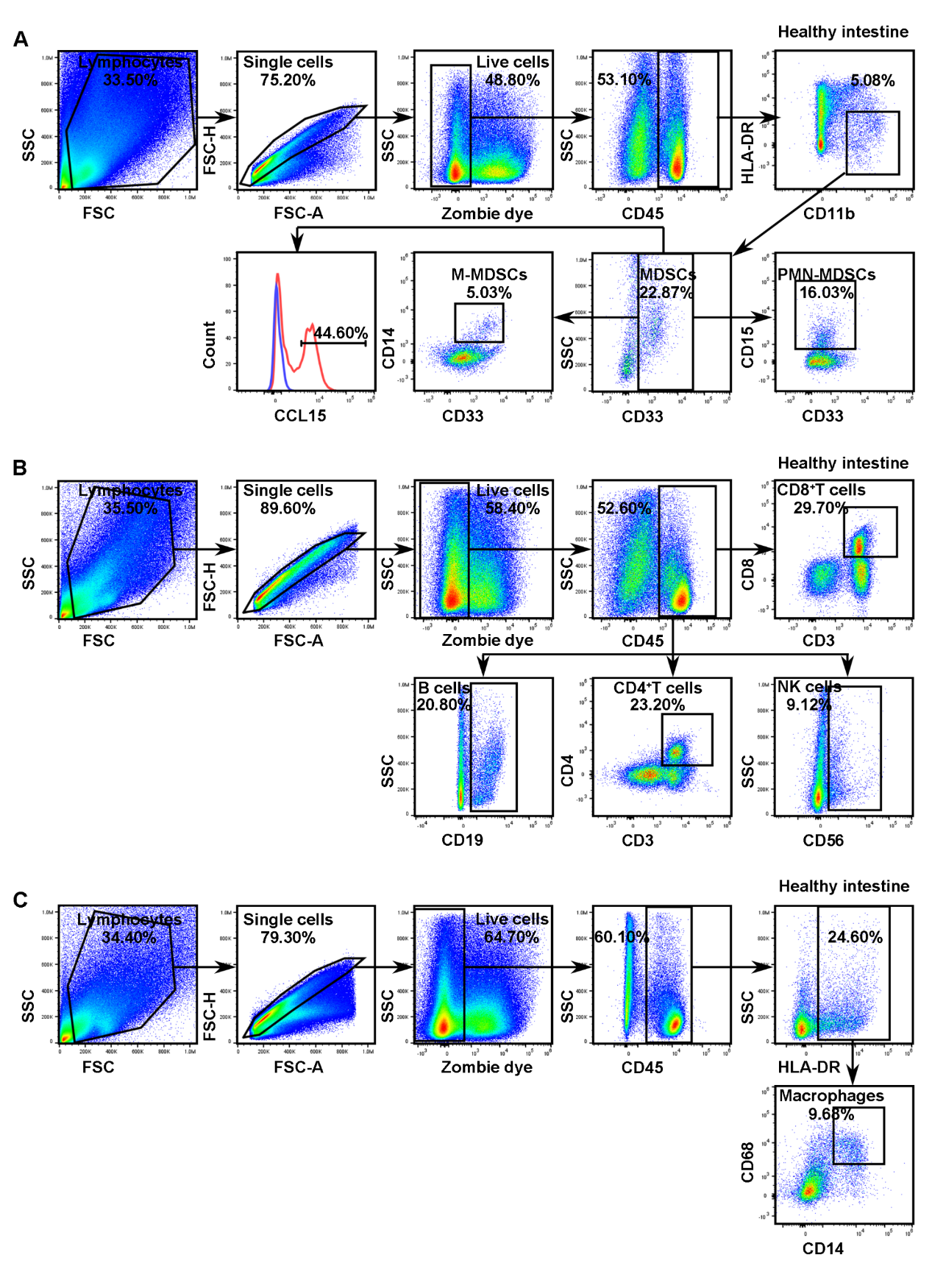


**Figure S5.** **Flow cytometry gating strategy for lamina propria immune cell populations in healthy human intestinal tissues.** A) MDSCs (CD11b^+^HLA-DR^-/low^CD33^+^), their subpopulations M-MDSCs (CD11b^+^HLA-DR^-/low^CD33^+^CD14^+^) and PMN-MDSCs (CD11b^+^HLA-DR^-/low^CD33^+^CD15^+^), and the expression of CCL15 (CCL15^+^) within MDSCs of CD45^+^ lamina propria immune cells from healthy human intestinal tissues were analyzed. B) Gating strategies of flow cytometry were employed to analyze B cells (CD19^+^), CD4^+^ T cells (CD3^+^CD4^+^), CD8^+^ T cells (CD3^+^CD8^+^), and NK cells (CD56^+^) among CD45^+^ lamina propria immune cells from healthy human intestinal tissues. C) Macrophages (HLA-DR^+^CD14^+^CD68^+^) within the CD45^+^ lamina propria immune cells of healthy human intestinal tissues were identified.


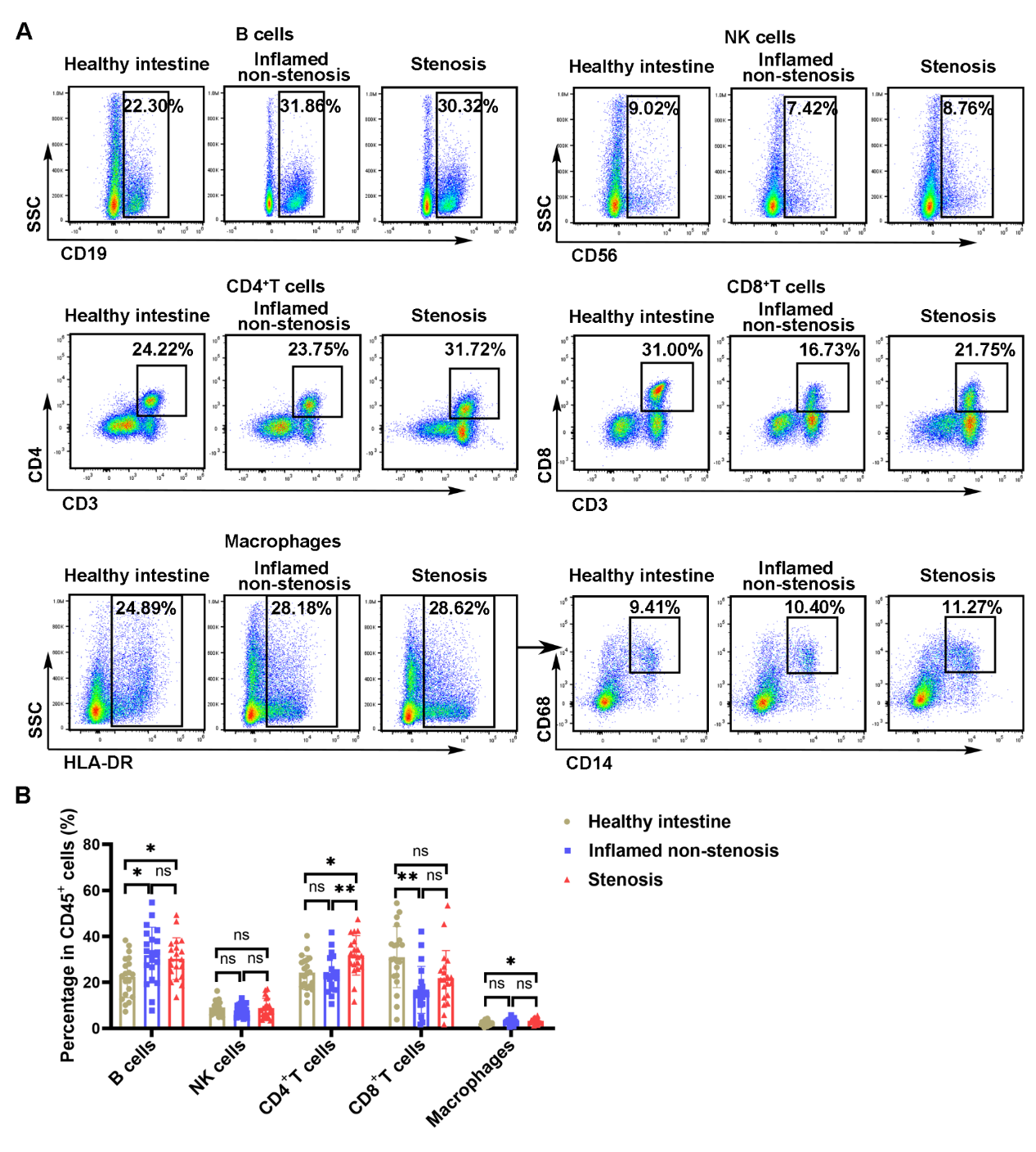


**Figure S6.** **Analysis of immune cell population proportions in the lamina propria of CD patients with intestinal stenosis.** A) Flow cytometry was used to analyze the proportions of B cells (CD19^+^), NK cells (CD56^+^), CD4^+^T (CD3^+^CD4^+^), CD8^+^T (CD3^+^CD8^+^), and macrophages (HLA-DR^+^CD14^+^CD68^+^) in healthy intestinal tissue, inflamed non-stenotic tissue, and stenotic tissue from CD patients with stenosis. B) Statistical analysis was performed. n = 20 patients. Data are presented as means ± SD. Statistical analyses were performed using one-way ANOVA with Bonferroni’s multiple comparison test. **P <* 0.05 and ***P <* 0.01, ns, not significant.


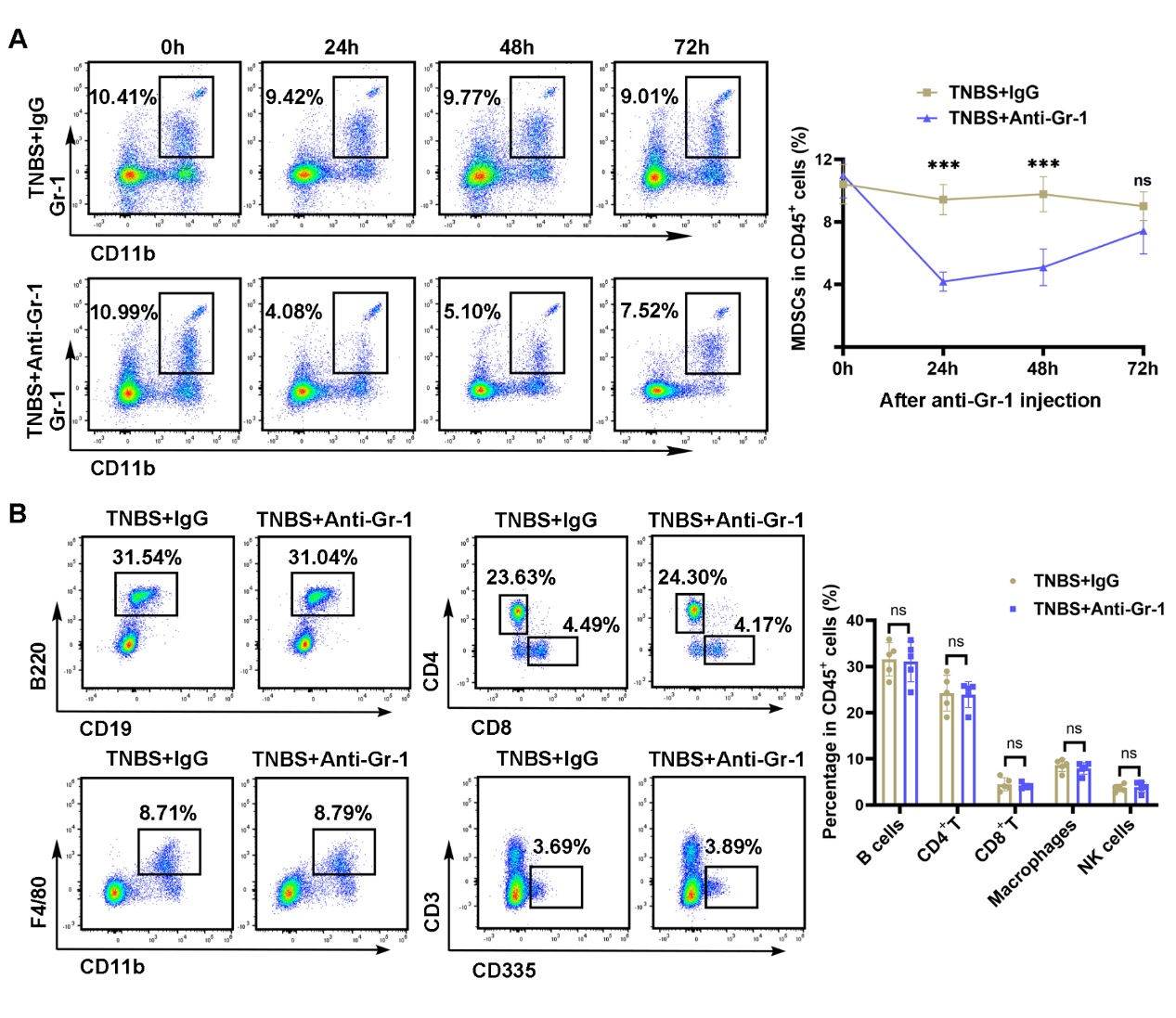


**Figure S7. *In vivo* validation of anti-Gr-1 antibody efficacy and specificity.** A) Mice received a 100 µL enema of 2% TNBS, followed 48 hours later by an intraperitoneal injection of 100 µg anti-Gr-1 antibody. MDSC levels in the colon were analyzed by flow cytometry at 24, 48, and 72-hours post-injection. B) Flow cytometry analyzed immune cell populations in colon tissues from treated mice. Representative flow cytometry scatter plots are presented. n = 5 mice per group. Data are presented as means ± SD. Statistical analysis for panel A used two-way ANOVA followed by Bonferroni’s multiple comparisons test, while panel B (CD4^+^ T cells) was analyzed using the Mann-Whitney U test, and other populations were assessed using unpaired Student’s t test (two tailed). ****P <* 0.001, ns, not significant.


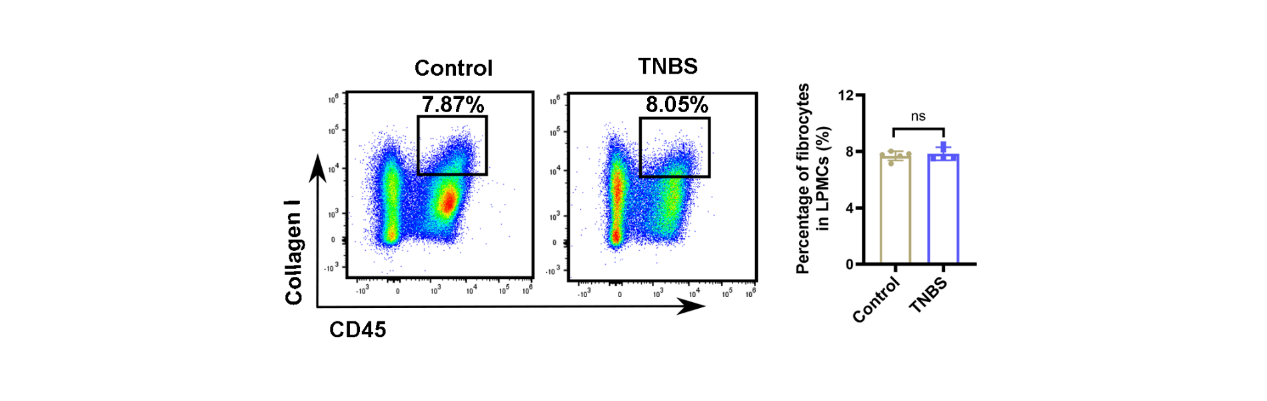


**Figure S8. Flow cytometry assessed fibrocyte proportions in colonic** **LPMCs from normal and TNBS-treated fibrotic mice.** This analysis was conducted to determine the contribution of fibrocytes to the development of intestinal fibrosis. n = 5 mice per group. Data are presented as means ± SD. Statistical analysis used unpaired Student’s t test (two tailed). ns, not significant.


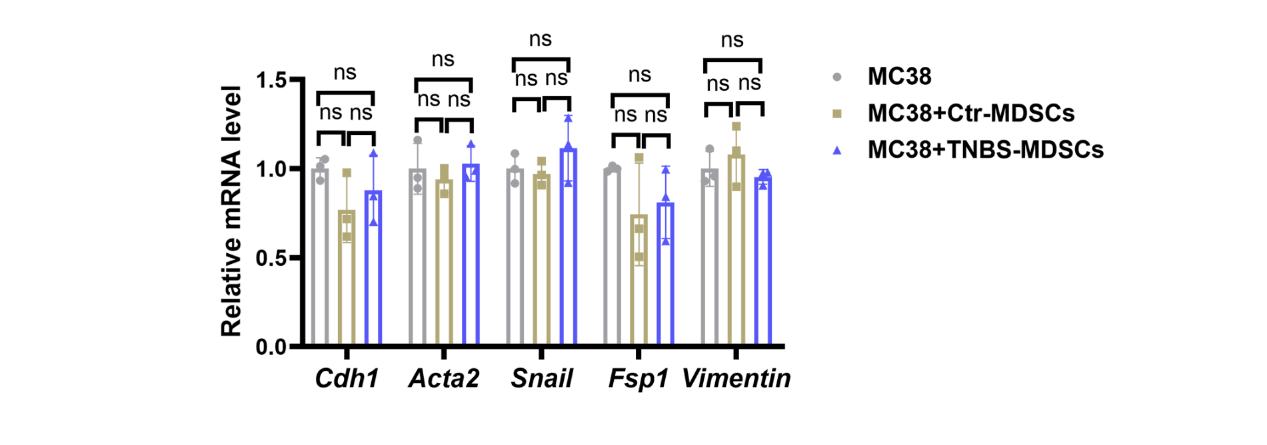


**Figure S9. qRT-PCR was conducted to evaluate the mRNA levels of *E-cadherin, Acta2, Snail, Fsp1, and Vimentin* in MC38 cells after co-culture with colonic MDSCs at a 1:10 ratio for 24 hours**. This analysis aimed to determine the role of epithelial-mesenchymal transition (EMT) in intestinal fibrosis. n = 3 biologically independent samples. Data are presented as means ± SD. Statistical analysis was performed using one-way ANOVA with Bonferroni’s post hoc test for multiple comparisons. ns, not significant.


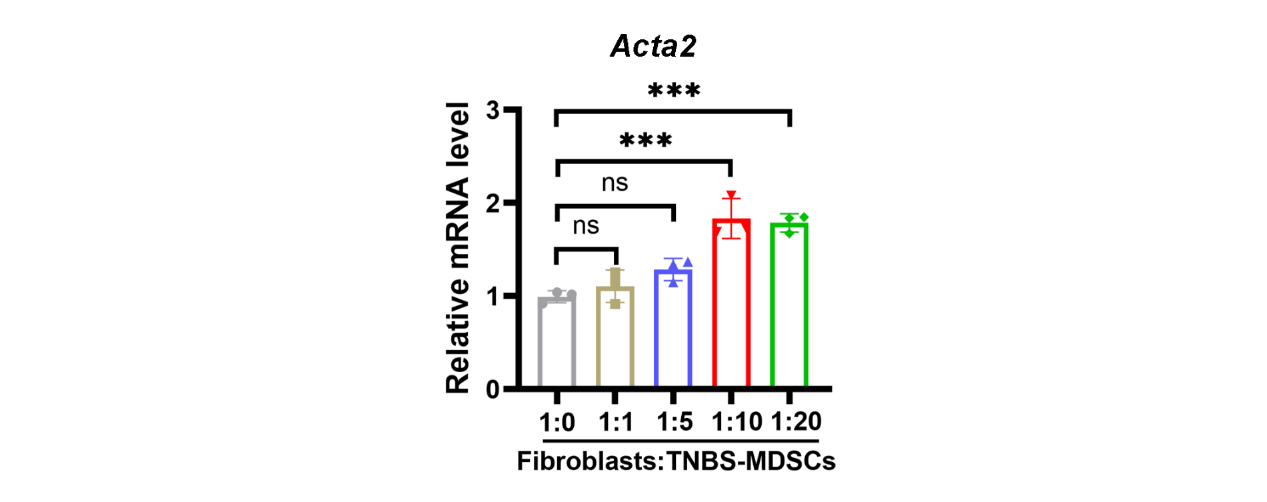


**Figure S10.** **Optimization of the co-culture ratio of colonic fibroblasts to MDSCs from mice with intestinal fibrosis.** Colonic fibroblasts and MDSCs, isolated from mice with TNBS-induced fibrosis, were co-cultured at varying ratios (1:1, 1:5, 1:10, and 1:20) for 24 hours. Fibroblast activation was assessed by quantifying Acta2 mRNA expression using qRT-PCR. n = 3 biologically independent samples. Data are represented as mean ± SD. Statistical analysis was performed using one-way ANOVA with Bonferroni’s post hoc test for multiple comparisons. ****P <* 0.001, ns, not significant.


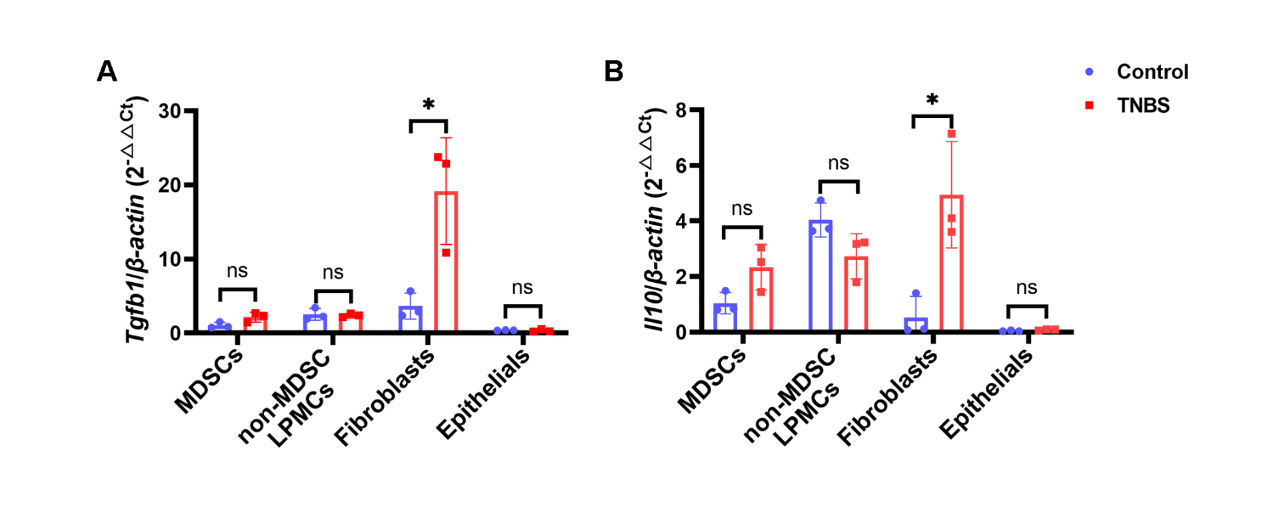


**Figure S11.** **qRT-PCR assessed the mRNA levels of A) *Tgfb1* and B) *Il10* in colonic MDSCs, non-MDSC LPMCs, fibroblasts, and epithelial cells from control and TNBS-treated mice.** n = 3 mice per group. Data are represented as mean ± SD. Statistical analysis used unpaired Student’s t test (two tailed). **P <* 0.05, ns, not significant.


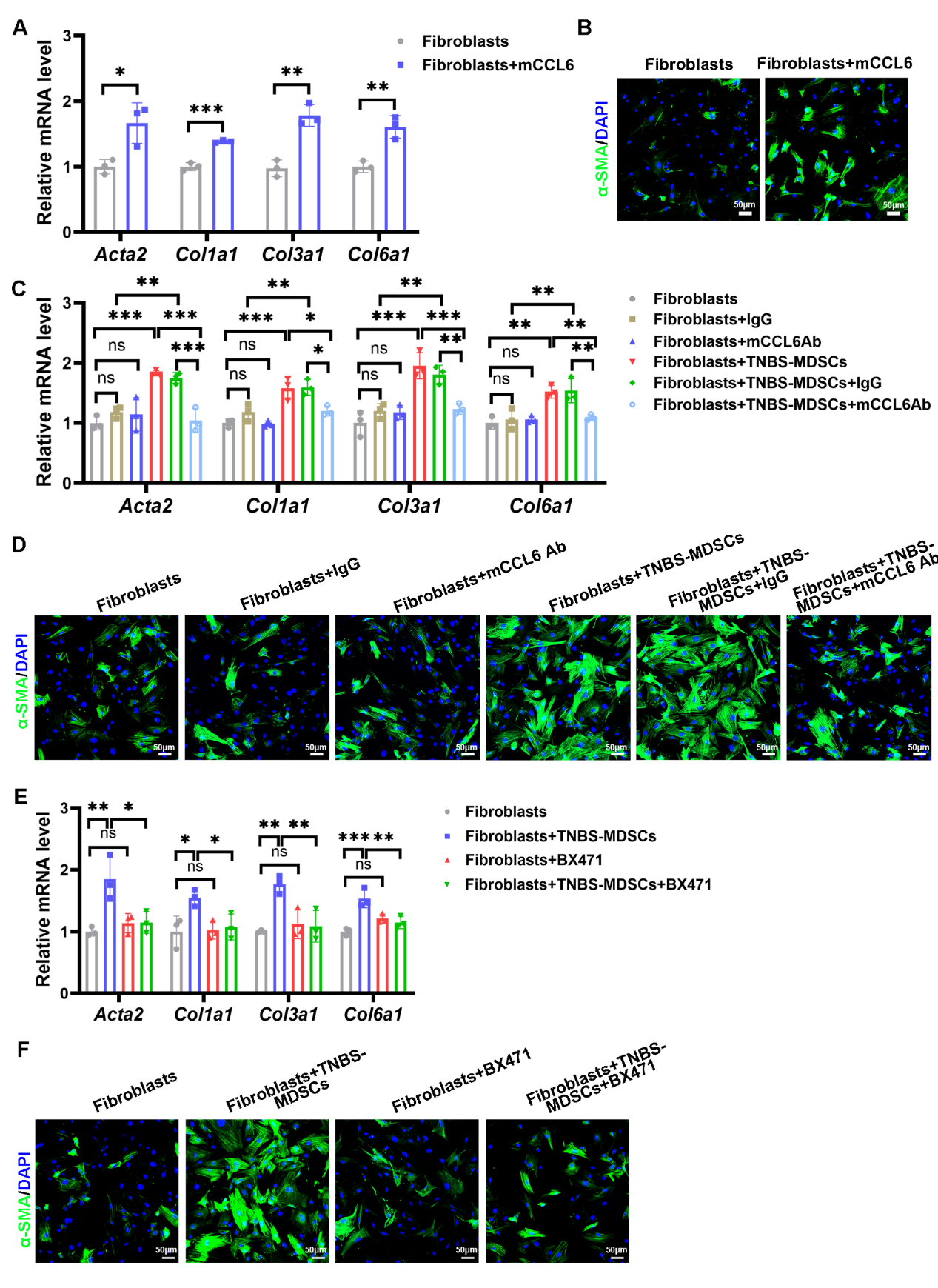


**Figure S12. Effects of the CCL6-CCR1 signaling pathway on fibroblast activation and collagen production.** A-B) Primary intestinal fibroblasts were treated with 25 ng/mL mCCL6 for 24 hours and analyzed by qRT-PCR for *Acta2*, *Col1a1*, *Col3a1* and *Col6a1* expression, and by immunofluorescence staining for α-SMA (Green) with DAPI nuclear staining (Blue). C-F) Primary intestinal fibroblasts were co-cultured with fibrotic colonic MDSCs at a 1:10 ratio for 24 hours, with or without mCCL6-neutralizing antibodies (40 ng/mL) or BX471 (20 μM). qRT-PCR and immunofluorescence were performed as above. Scale bars indicate reference measurements. n = 3 biologically independent samples for panels A, C, E. Experiments were repeated three times for panel B, D, F with consistent results. Data are presented as means ± SD. Statistical analysis for panel A used unpaired Student’s t test (two tailed), while panels C, E were analyzed using one-way ANOVA with Bonferroni’s post hoc test for multiple comparisons. **P <* 0.05, ***P <* 0.01 and ****P <* 0.001, ns, not significant.


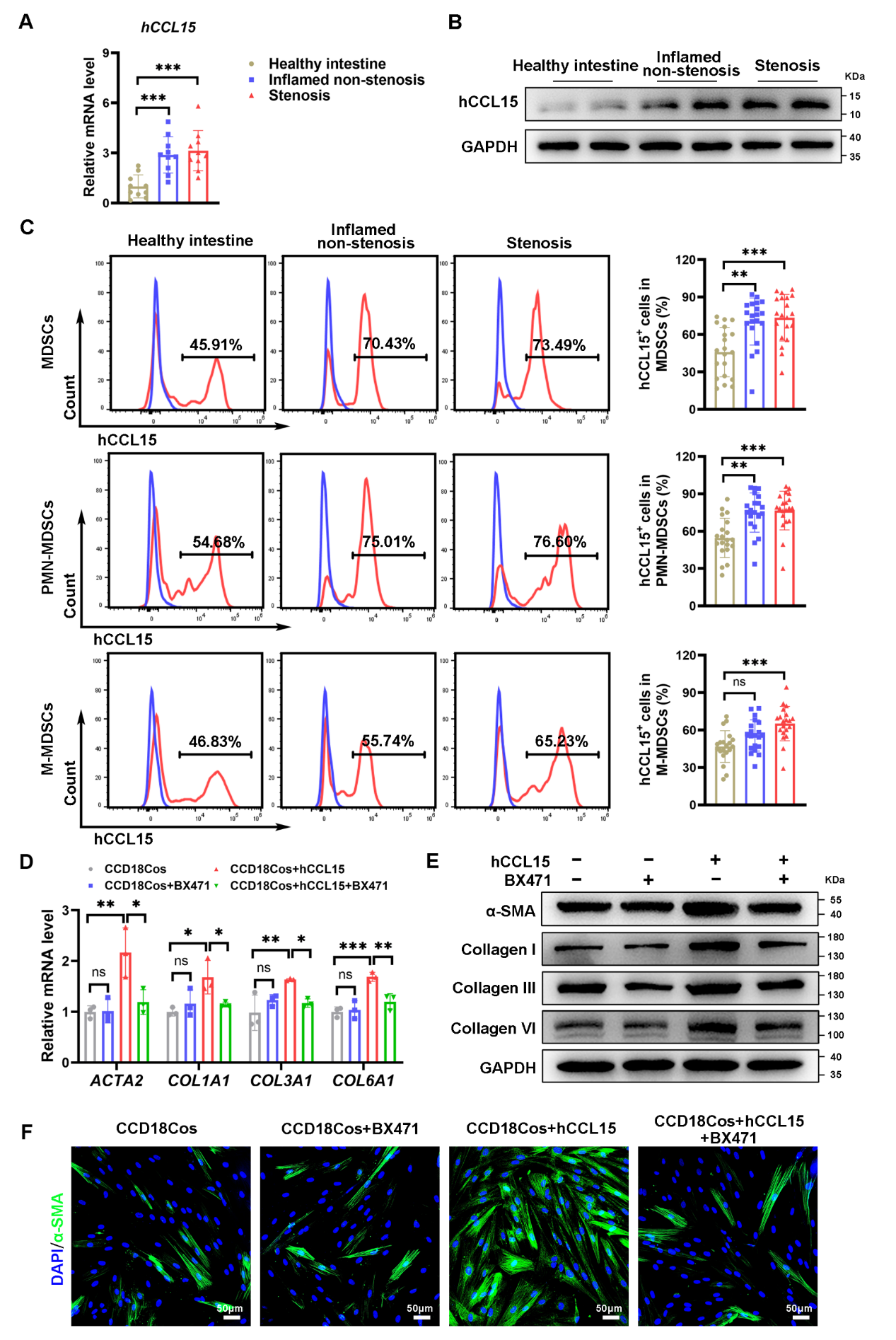


**Figure S13. Analysis of hCCL15 expression in CD patients with intestinal stenosis and its effects on human fibroblasts.** A-B) qRT-PCR and Western blotting analysis were conducted to assess *hCCL15* mRNA levels in healthy intestine, inflamed non-stenotic tissue, and stenotic tissue from CD patients with stenosis (n = 10 for Panel A and n = 2 for Panel B). C) hCCL15 expression was evaluated in MDSCs, PMN-MDSCs, and M-MDSCs from different intestinal segments of CD patients with stenosis (n = 20). D-F) The human intestinal fibroblast cell line CCD18Co was starved for 6 hours and then incubated with cytokine hCCL15 for 24 hours. In some experiments, CCD18Co cells were pre-treated with BX471 (20 μM) 6 hours prior to hCCL15 stimulation and throughout the stimulation period. The expression levels of α-SMA and collagen (types I, III and VI) were measured by D) qRT-PCR, E) Western blotting and F) immunofluorescence staining (α-SMA in green; DAPI nuclear staining in blue; scale bar for reference). Results represent three biologically independent samples for panel D and were obtained from three independent experiments for panels F. Data are presented as means ± SD. Statistical analyses were performed using Kruskal-Wallis test with Dunn’s multiple comparisons test for panel C (hCCL15 expression in MDSCs and PMN-MDSCs), and one-way ANOVA with Bonferroni’s multiple comparison test for the remaining panels. **P <* 0.05, ***P <* 0.01 and ****P <* 0.001, ns, not significant.


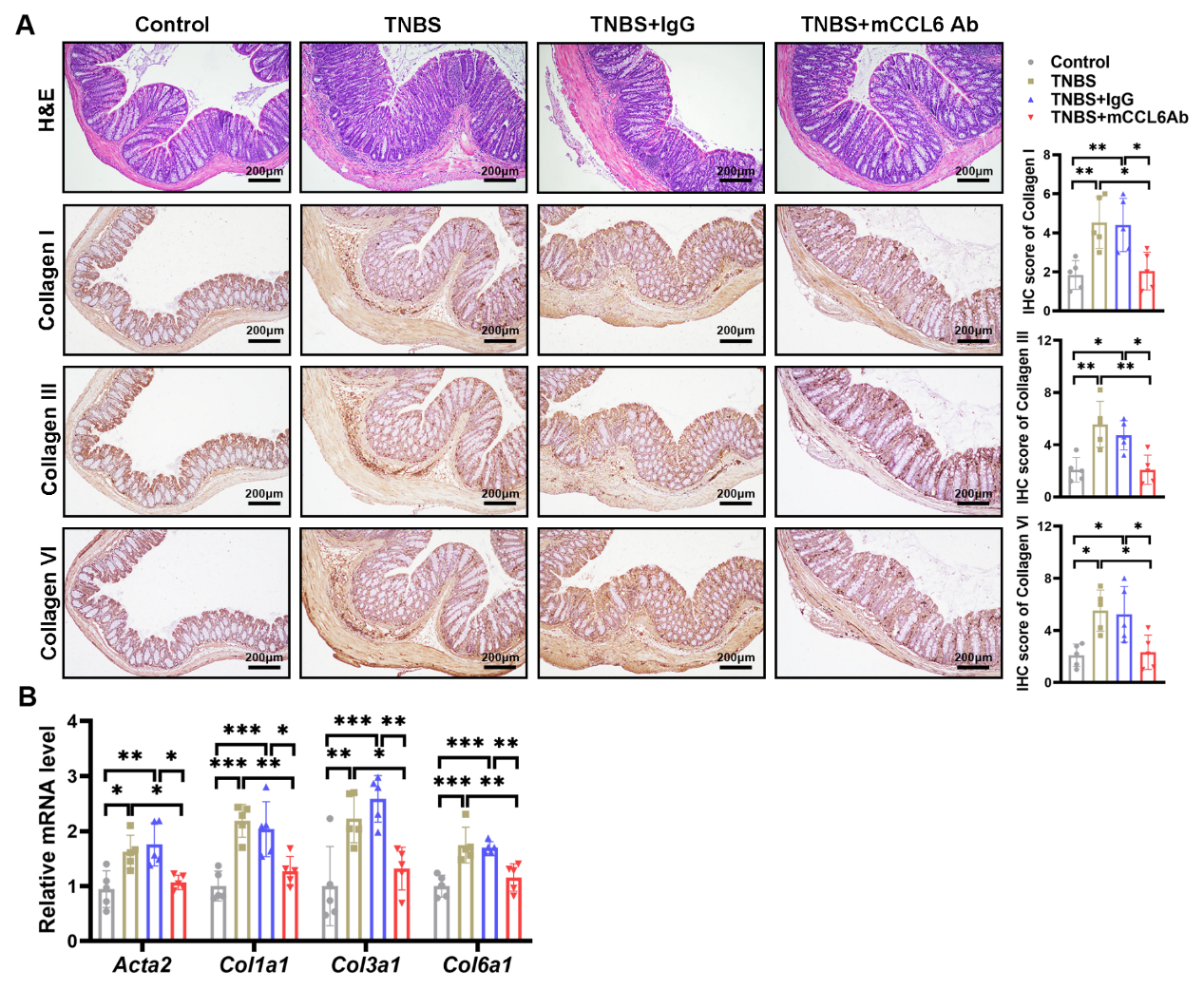


**Figure S14. Treatment with an anti-murine CCL6-neutralizing antibody alleviated the symptoms of intestinal fibrosis in TNBS-treated mice.** A) Histopathological evaluations, including H&E staining and immunohistochemical staining for collagen types I, III, and IV, were conducted on serial tissue sections. A scale bar is provided for reference. Tissue sections from each mouse were examined, with five fields captured for quantitative analysis; each data point represents the mean of these fields. Representative images are presented. IHC staining intensities were quantified using ImageJ software. B) Colon tissues were collected for qRT-PCR to assess the expression of *Acta2*, *Col1a1*, *Col3a1*, and *Col6a1*. n = 5 mice per group. Data are presented as means ± SD. Statistical analyses were performed using one-way ANOVA with Bonferroni’s post hoc test for multiple comparisons. **P <* 0.05, ***P <* 0.01 and ****P <* 0.001.


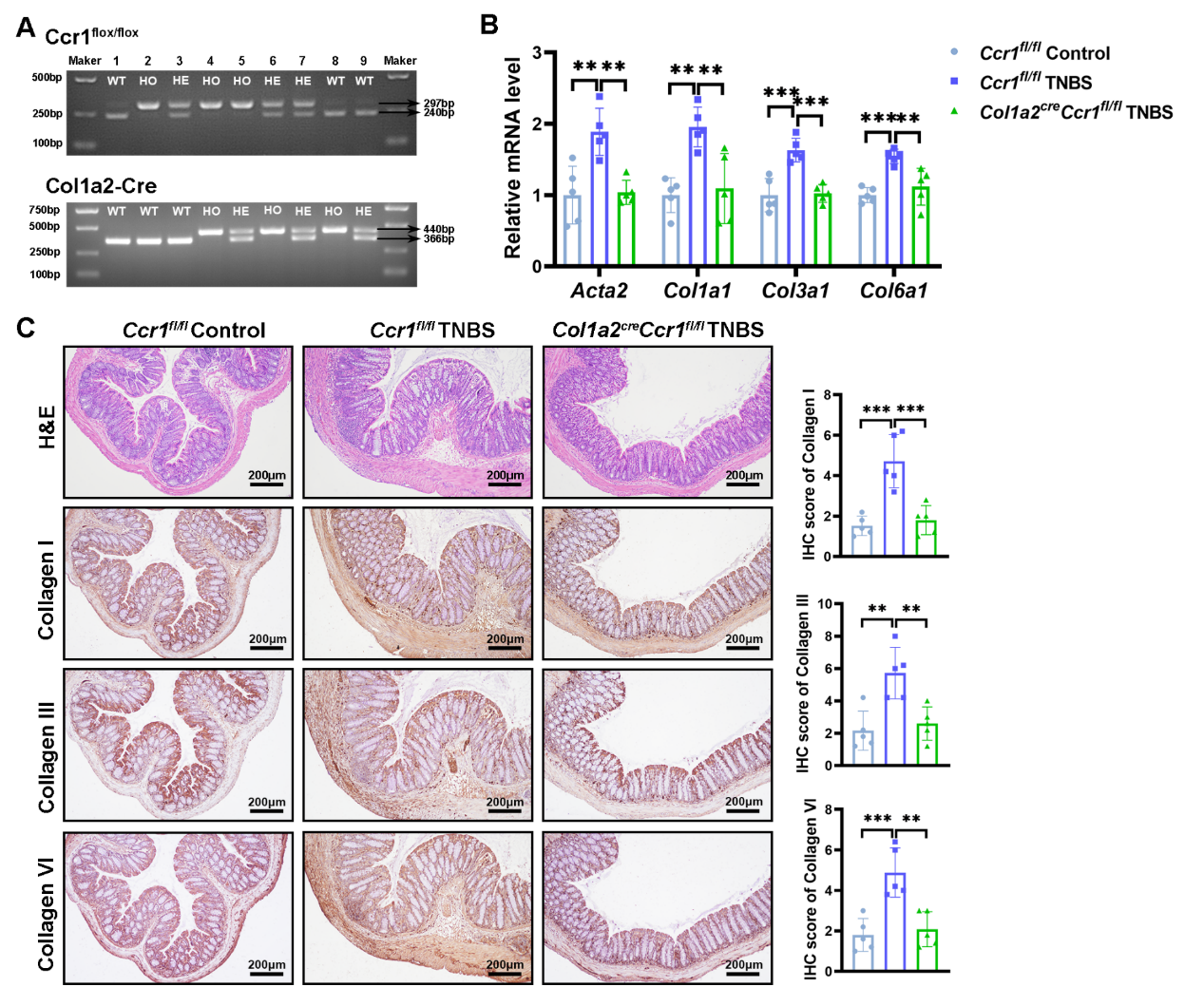


**Figure S15.** **Deletion of the *Ccr1* gene specifically in fibroblasts attenuated the severity of intestinal fibrosis in TNBS-induced mice.** A) Genotyping was performed by mouse tail DNA, PCR amplification and agarose gel electrophoresis. *Ccr1^fl/fl^* wild type (WT): 240 bp, heterozygote (HE): 297 bp and 240 bp, homozygote (HO): 297 bp; *Col1a2^cre^* WT: 366 bp, HE: 366 bp and 440 bp, HO: 440 bp. Mice numbered 4 and 5 were identified as fibroblast-specific *Ccr1* knockout mice. B) Colon tissues were collected for qRT-PCR to assess the expression of *Acta2*, *Col1a1*, *Col3a1*, and *Col6a1*. n = 5 mice per group. C) Histopathological evaluations, including H&E staining and immunohistochemical staining for collagen types I, III, and VI, were conducted on serial tissue sections. Tissue sections from each mouse were examined, with five random fields captured for quantitative analysis; each data point represents the mean of these fields. Representative images are presented. IHC staining intensities were evaluated using ImageJ software. Data are presented as means ± SD. Statistical analyses were performed using one-way ANOVA with Bonferroni’s post hoc test multiple comparisons. **P <* 0.05, ***P <* 0.01 and ****P <* 0.001.


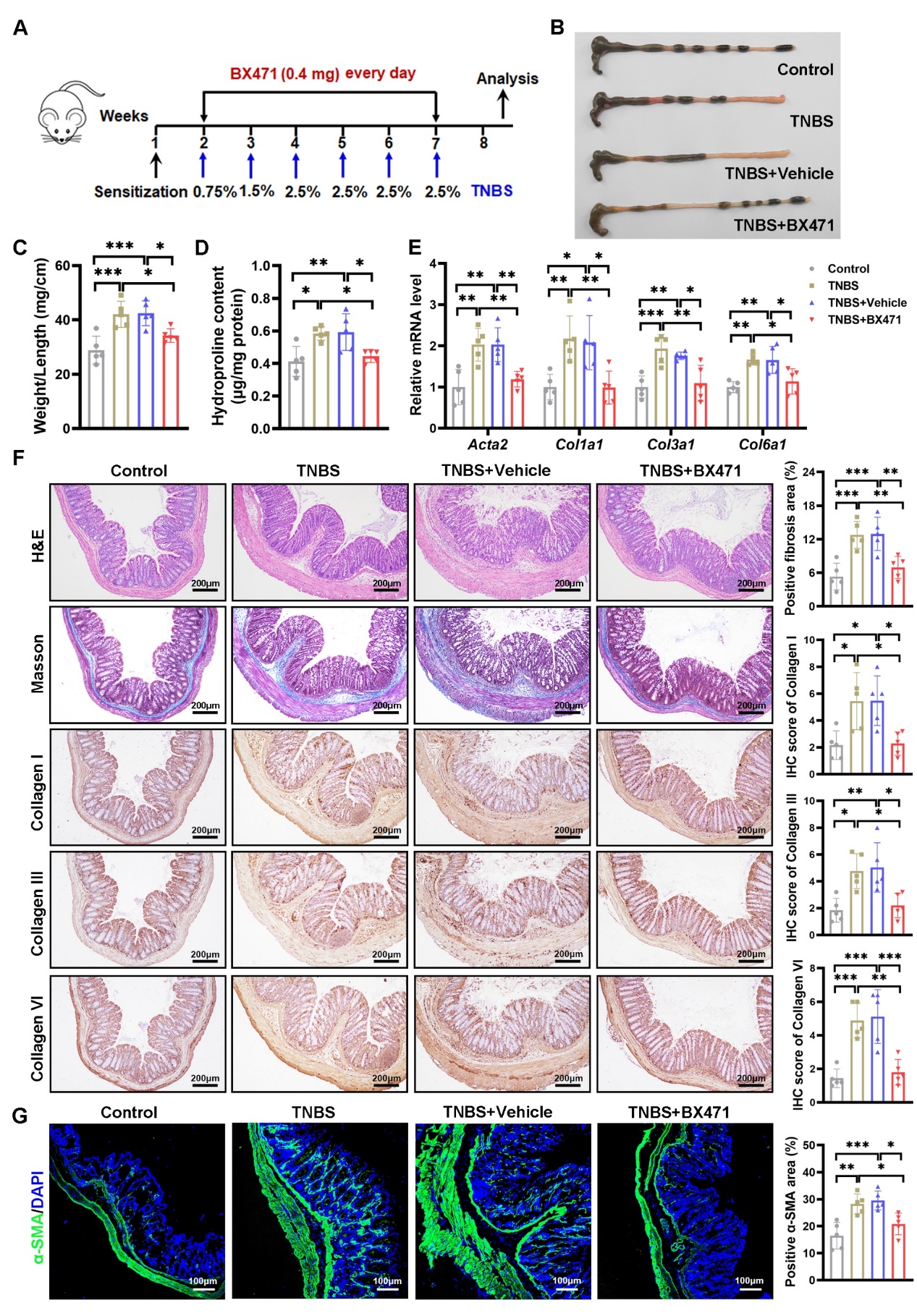


**Figure S16.** **Treatment with BX471 mitigated symptoms of intestinal fibrosis in TNBS-treated mice.** A) The experimental design outlines the methodology for assessing the therapeutic effects of BX471 on TNBS-induced intestinal fibrosis mice. B-E) Colon tissues were collected for a range of assessments, including B) macroscopic observation, C) calculation of weight-to-length ratio, D) hydroxyproline content assays, and E) qRT-PCR for *Acta2*, *Col1a1*, *Col3a1*, and *Col6a1*. F-G) Histopathological evaluations were performed on serial tissue sections, including F) H&E staining and Masson's trichrome staining, immunohistochemical staining for collagen types I, III, and VI，as well as G) immunofluorescence staining for α-SMA (α-SMA in green; DAPI nuclear staining in blue). A scale bar is provided for reference. Tissue sections from each mouse were examined, with five random fields captured for quantitative analysis, and each data point represents the mean of these fields. Representative images are presented. The average percentages of Masson's trichrome and α-SMA positive areas，as well as the IHC staining scores, were quantified using ImageJ software. n = 5 mice per group. Data are presented as means ± SD. Statistical analyses were performed using one-way ANOVA with Bonferroni’s post hoc test for multiple comparisons. **P <* 0.05, ***P <* 0.01 and ****P <* 0.001.


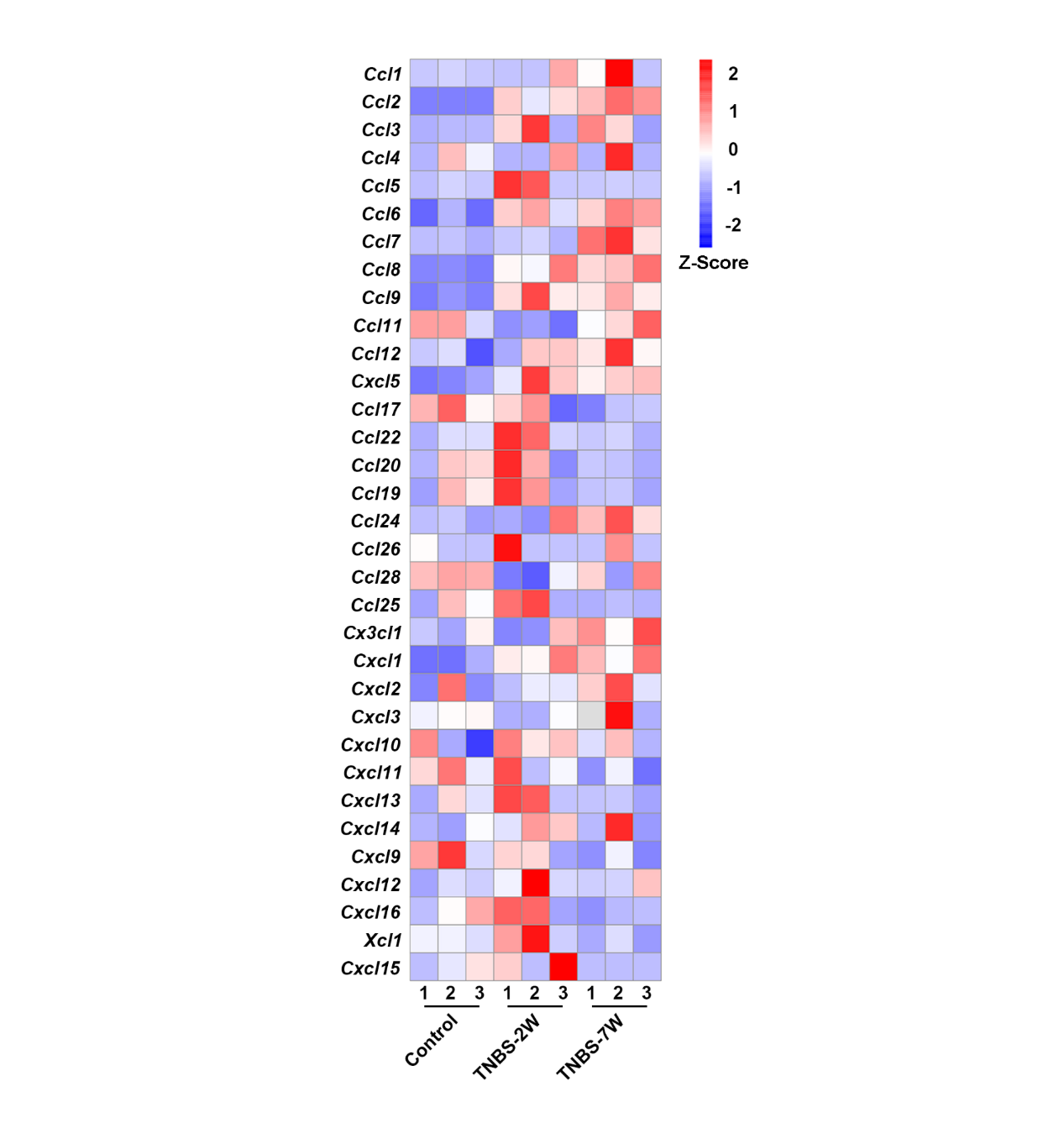


**Figure S17.** **Profiling chemokine gene expression in the colon of mice with intestinal fibrosis.** A chemokine PCR array was used to assess colonic chemokine gene expression in control healthy mice and in TNBS-induced intestinal fibrosis models at distinct time points (2 and 7 weeks post-induction). n = 3 mice per group. Data analysis was performed using Wcgene Biotech software and the results were visualized as heat maps created with HemI (heatmap Illustrator, version 1.0).


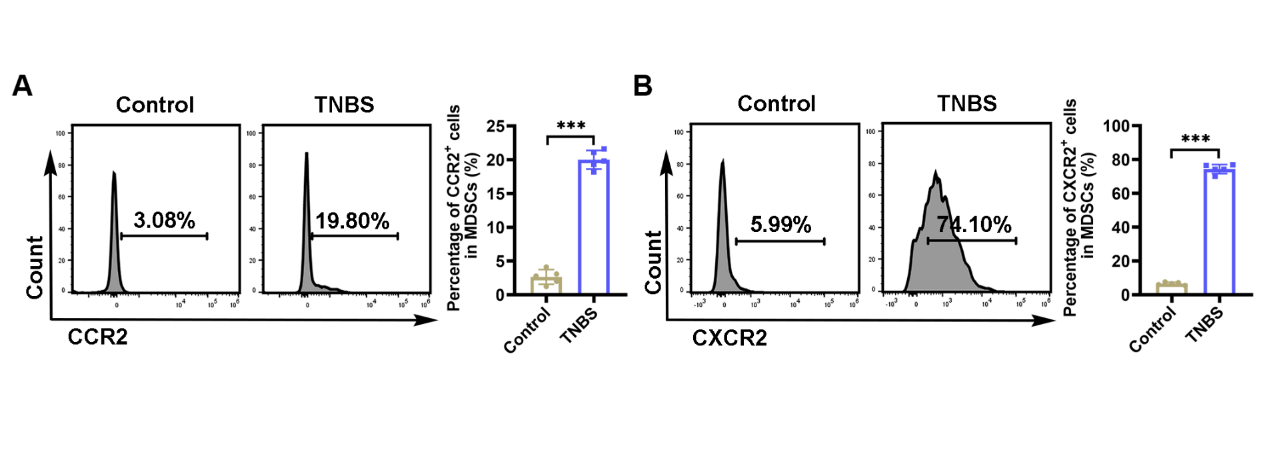


**Figure S18. Assessment of CCR2 and CXCR2 receptor expression on MDSCs in a murine model of intestinal fibrosis.** (A) CCR2 and (B) CXCR2 receptor expression on MDSCs within the colonic lamina propria of control and intestinal fibrosis mice were determined by flow cytometry. n = 5 mice per group. Data are presented as means ± SD. Statistical analyses were performed using unpaired Student’s t test (two tailed). ****P <* 0.001.


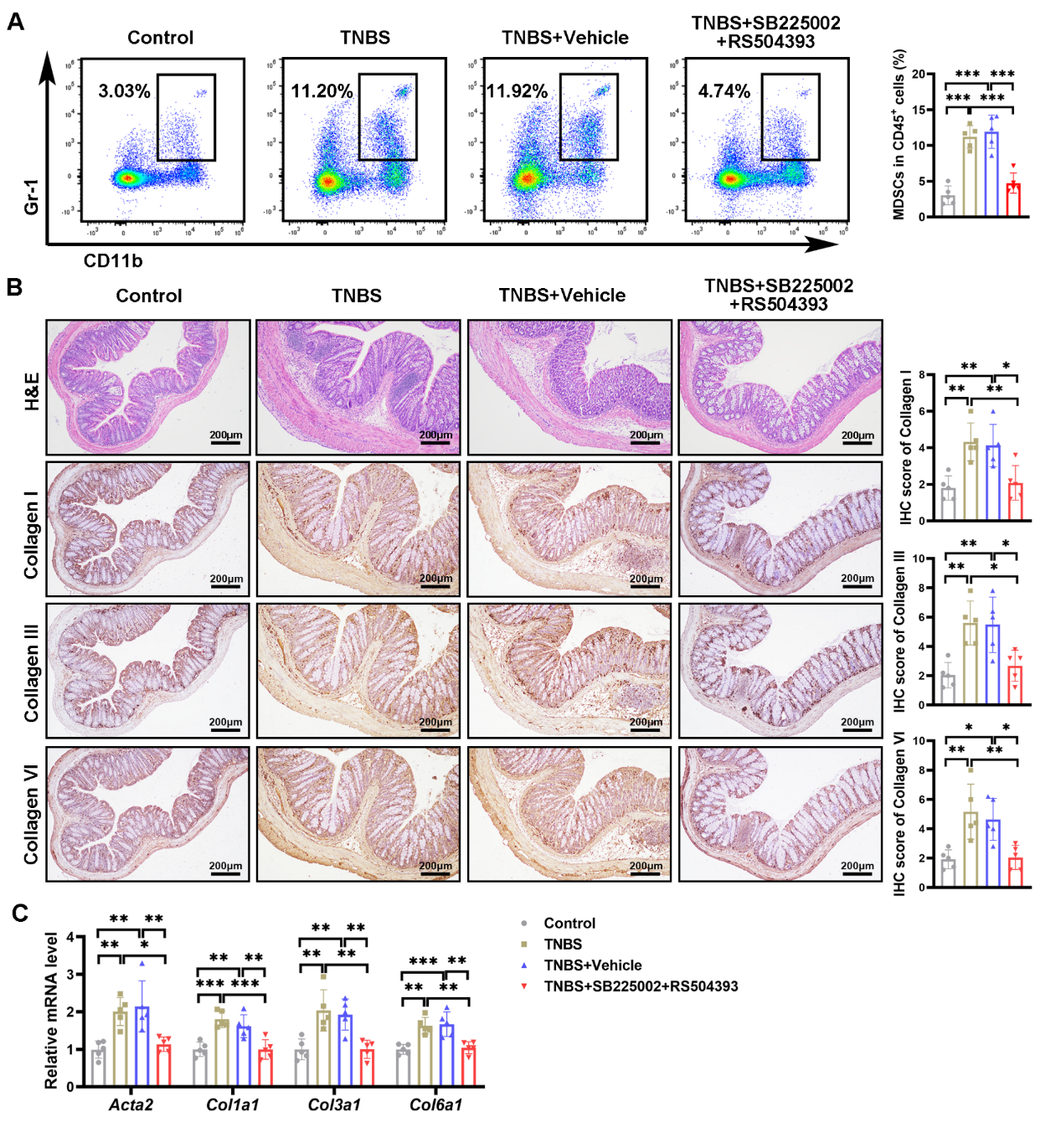


**Figure S19. Combined treatment with** **SB225002 and RS504393 inhibited MDSCs recruitment and ameliorated intestinal fibrosis in TNBS-treated mice.** A) Flow cytometry was conducted to evaluate the MDSCs population among colonic lamina propria cells in mice subjected to various treatment conditions. B) Histopathological evaluations were conducted on serial tissue sections, including H&E staining, and immunohistochemical staining for collagen types I, III, and VI. IHC staining intensities were quantified using ImageJ software. A scale bar is provided for reference. Tissue sections from each mouse were examined, and five fields were captured for quantitative analysis, with each data point representing the mean of these fields. C) Colon tissues were collected for qRT-PCR to evaluate the expression of *Acta2*, *Col1a1*, *Col3a1*, and *Col6a1*. n = 5 mice per group. Data are presented as means ± SD. Statistical analyses were performed using one-way ANOVA with Bonferroni’s post hoc test for multiple comparisons. **P <* 0.05, ***P <* 0.01 and ****P <* 0.001.

**Table S1.** Clinical Characteristics of CD Patients with Intestinal Stenosis.

| Group | CD with Intestinal stenosis |
| --- | --- |
| Number of patients | 40 |
| Age(y) |  |
| 15-40 years | 21 |
| >40 years | 19 |
| Gender |  |
| Female | 17 |
| Male | 23 |

**Table S2.** Antibodies Used for Flow Cytometry (FC), Western Blotting (WB), Immunohistochemistry (IHC) and Immunofluorescence (IF) in This Study.

| Antibody | Catalogue No. | Manufacturer | Concentration | Application |
| --- | --- | --- | --- | --- |
| APC anti-mouse CD45 | 103112 | Biolegend | 1:100 | FC |
| PE/Cy7 anti-mouse CD11b | 101216 | Biolegend | 1:100 | FC |
| PE anti-mouse Gr-1 | 108408 | Biolegend | 1:100 | FC |
| BV605 anti-mouse Ly6G | 127639 | Biolegend | 1:100 | FC |
| BV711 anti-mouse Ly6C | 128037 | Biolegend | 1:50 | FC |
| BV421 anti-mouse F4/80 | 123137 | Biolegend | 1:100 | FC |
| FITC anti-mouse CCL6 | orb13833 | Biorbyt | 1:50 | FC |
| FITC anti-mouse CD45 | 103108 | Biolegend | 1:200 | FC |
| BV605 anti-mouse CD3 | 100237 | Biolegend | 1:20 | FC |
| BV711 anti-mouse CD4 | 100447 | Biolegend | 1:100 | FC |
| PE/Cy7 anti-mouse CD8 | 100722 | Biolegend | 1:100 | FC |
| BV421 anti-mouse CD335 | 137611 | Biolegend | 1:50 | FC |
| APC anti-mouse CD19 | 115512 | Biolegend | 1:100 | FC |
| PE anti-mouse B220 | 103207 | Biolegend | 1:100 | FC |
| Antibody | Catalogue No. | Manufacturer | Concentration | Application |
| APC/Cy7 anti-mouse CXCR2 | 149313 | Biolegend | 1:20 | FC |
| BV510 anti-mouse CCR2 | 150617 | Biolegend | 1:50 | FC |
| APC anti-mouse CD25 | 101910 | Biolegend | 1:100 | FC |
| PE anti-mouse Foxp3 | 126404 | Biolegend | 1:100 | FC |
| BV510 anti-human CD45 | 563204 | BD Biosciences | 1:20 | FC |
| APC anti-mouse/human CD11b | 101212 | Biolegend | 1:100 | FC |
| PE anti-human CD33 | 303404 | Biolegend | 1:20 | FC |
| BV711 anti-human CD14 | 301837 | Biolegend | 1:20 | FC |
| BV605 anti-human CD15 | 323031 | Biolegend | 1:20 | FC |
| FITC anti-human CCL15 | orb415075 | Cisbio | 1:50 | FC |
| APC anti-human CD11c | 301614 | Biolegend | 1:20 | FC |
| FITC anti-human CD3 | 300306 | Biolegend | 1:20 | FC |
| BV605 anti-human CD4 | 300555 | Biolegend | 1:20 | FC |
| BV711 anti-human CD8 | 344733 | Biolegend | 1:20 | FC |
| Antibody | Catalogue No. | Manufacturer | Concentration | Application |
| PE anti-human CD56 | 555516 | BD Biosciences | 1:20 | FC |
| FITC anti-human CD14 | 301804 | Biolegend | 1:20 | FC |
| APC anti-human CD68 | 333810 | Biolegend | 1:20 | FC |
| AF 700 anti-human HLA-DR | 307626 | Biolegend | 1:25 | FC |
| anti-α-SMA | BM0002 | Boster | 1:500  1:1000 | WB  IHC/IF |
| anti-Collagen Type Ⅰ | BA0325 | Boster | 1:500  1:200 | WB  IHC |
| anti-Collagen Type Ⅲ | A00788-3 | Boster | 1:300 | IHC |
| anti-Collagen Type Ⅲ | 22734-1-AP | Proteintech | 1:500 | WB |
| anti-Collagen Type Ⅵ | 17023-1-AP | Proteintech | 1:1000  1:300 | WB  IHC |
| anti-p-ERK1/2 | 4370T | Cell Signaling Technology | 1:2000 | WB |
| anti-ERK1/2 | ab184699 | Abcam | 1:10000 | WB |
| anti-p-p38 | 4511T | Cell Signaling Technology | 1:1000 | WB |
| anti-p38 | 8690T | Cell Signaling Technology | 1:1000 | WB |
| anti-CCL6 | ab275025 | Abcam | 1:1000 | WB |
| anti-CCL15 | orb32335 | Biorbyt | 1:1000 | WB |
| HRP-conjugated anti-mouse IgG | 115-035-044 | Jackson ImmunoResearch | 1:2000 | WB |
| Antibody | Catalogue No. | Manufacturer | Concentration | Application |
| HRP-conjugated anti-rabbit IgG | 111-035-003 | Jackson ImmunoResearch | 1:2000 | WB |
| HRP conjugated anti-GAPDH | HRP-60004 | Proteintech | 1:10000 | WB |
| rabbit anti-CD11b | ab133357 | Abcam | 1:2000 | IF |
| rat anti-CD11b | 101202 | Biolegend | 1:100 | IF |
| rabbit anti-CD33 | ab269456 | Abcam | 1:50 | IF |
| rat anti-Gr-1 | 108402 | Biolegend | 1:100 | IF |
| Alexa Fluor 488 donkey  anti-mouse IgG | A21202 | Invitrogen | 1:200 | IF |
| Alexa Fluor 488 donkey anti-rat IgG | A21208 | Invitrogen | 1:200 | IF |
| Alexa Fluor 647 goat anti-rabbit IgG | A21244 | Invitrogen | 1:200 | IF |
| Alexa Fluor 546 Goat anti-Rat IgG | A21247 | Invitrogen | 1:200 | IF |

**Table S3.** Primers Used for PCR in This Study.

| Name of primer | Sequence |
| --- | --- |
| Mouse *β-actin*-Forward | GTGAAAAGATGACCCAGATCAT |
| Mouse *β-actin*-Reverse | GCTTCTCTTTGATGTCACGCACGAT |
| Mouse *Acta2*-Forward | CCCAACTGGGACCACATGG |
| Mouse *Acta2*-Reverse | TACATGCGGGGGACATTGAAG |
| Mouse *Col1a1*-Forward | CATAAAGGGTCATCGTGGCT |
| Mouse *Col1a1*-Reverse | TTGAGTCCGTCTTTGCCAG |
| Mouse *Col3a1*-Forward | GAAGTCTCTGAAGCTGATGGG |
| Mouse *Col3a1*-Reverse | TTGCCTTGCGTGTTTGATATTC |
| Mouse *Col6a1*-Forward | CTGCTGCTACAAGCCTGCT |
| Mouse *Col6a1*-Reverse | CCCCATAAGGTTTCAGCCTCA |
| Mouse *Ccl6*-Forward | GCTGGCCTCATACAAGAAATGG |
| Mouse *Ccl6*-Reverse | GCTTAGGCACCTCTGAACTCT |
| Mouse *Ccr1*-Forward | CTCATGCAGCATAGGAGGCTT |
| Mouse *Ccr1*-Reverse | ACATGGCATCACCAAAAATCCA |
| Mouse *Cdh5*-Forward | CAGTTCCGAGGTCTACACCTT |
| Mouse *Cdh5*-Reverse | TGAATCGGGAGTCTTCCGAAAA |
| Mouse *Snail*-Forward | CACACGCTGCCTTGTGTCT |
| Mouse *Snail*-Reverse | GGTCAGCAAAAGCACGGTT |
| Mouse *Fsp1*-Forward | TGAGCAACTTGGACAGCAACA |
| Mouse *Fsp1*-Reverse | TTCCGGGGTTCCTTATCTGGG |
| Mouse *Vimentin*-Forward | TTTCTCTGCCTCTGCCAAC |
| Mouse *Vimentin*-Reverse | TCTCATTGATCACCTGTCCATC |
| Mouse *ll10*-Forward | GCTCTTACTGACTGGCATGAG |
| Mouse *ll10*-Reverse | CGCAGCTCTAGGAGCATGTG |
| Mouse *Tgfb1*-Forward | CTCCCGTGGCTTCTAGTGC |
| Mouse *Tgfb1*-Reverse | GCCTTAGTTTGGACAGGATCTG |
| Human *ACTB*-Forward | GACTGCTGTCACCTTCACCGTTC |
| Name of primer | Sequence |
| Human *ACTB*-Reverse | GACTTAGTTGCGTTACACCCTTTCTTG |
| Human *ACTA2*-Forward | CTATGAGGGCTATGCCTTGCC |
| Human *ACTA2*-Reverse | GCTCAGCAGTAGTAACGAAGGA |
| Human *C­­OL1A1*-Forward | GAGGGCCAAGACGAAGACATC |
| Human *C­­OL1A1*-Reverse | CAGATCACGTCATCGCACAAC |
| Human *C­­OL3A1*-Forward | TTGAAGGAGGATGTTCCCATCT |
| Human *C­­OL3A1*-Reverse | ACAGACACATATTTGGCATGGTT |
| Human *C­­OL6A1*-Forward | AGGAGTCAAAGGAGCAAAGG |
| Human *C­­OL6A1*-Reverse | GCATTCACAGCAAGAGCAC |
| Human *CCL15*-Forward | CTGACTGCTGCACCTCCTACATC |
| Human *CCL15*-Reverse | GACCACTGGGTTTGGCACAGAC |
| Human *CXCL5*-Forward | AGCTGCGTTGCGTTTGTTTAC |
| Human *CXCL5*-Reverse | TGGCGAACACTTGCAGATTAC |
| Human *CCL2*-Forward | CAGCCAGATGCAATCAATGCC |
| Human *CCL2*-Reverse | TGGAATCCTGAACCCACTTCT |
| Human *CXCL1*-Forward | CAAACCGAAGTCATAGCCACAA |
| Human *CXCL1*-Reverse | CTCCTAAGCGATGCTCAAACA |
| Human *CCL8*-Forward | TGGAGAGCTACACAAGAATCACC |
| Human *CCL8*-Reverse | TGGTCCAGATGCTTCATGGAA |
| Mouse *Col1a2^cre^*-Forward | GGTGCAGCTATACTGGTCTGA |
| Mouse *Col1a2^cre^*-Reverse1 | CAATCCTTCTCTTTTGCCCACA |
| Mouse *Col1a2^cre^*-Reverse2 | GCACACAGACAGGAGCATCT |
| Mouse *Ccr1*^Flox^-Forward | TCAGAGCTGGGATGGAACAAC |
| Mouse *Ccr1*^Flox^-Reverse | CAACTCCAAACAGCCGAGTT |
